# Associations of essential metals with the risk of aortic arch calcification: a cross‐sectional study in a mid‐aged and older population of Shenzhen, China

**DOI:** 10.1002/mco2.533

**Published:** 2024-05-13

**Authors:** Mingxing Mo, Li Yin, Tian Wang, Ziquan Lv, Yadi Guo, Jiangang Shen, Huanji Zhang, Ning Liu, Qiuling Wang, Suli Huang, Hui Huang

**Affiliations:** ^1^ Department of Cardiology Joint Laboratory of Guangdong‐Hong Kong‐Macao Universities for Nutritional Metabolism and Precise Prevention and Control of Major Chronic Diseases the Eighth Affiliated Hospital of Sun Yat‐sen University Shenzhen China; ^2^ School of Public Health Shenzhen University Medical School Shenzhen University Shenzhen Guangdong China; ^3^ Department of Central Laboratory Shenzhen Center for Disease control and Prevention Shenzhen China; ^4^ School of Chinese Medicine Li Ka Shing Faculty of Medicine The University of Hong Kong Hong Kong SAR China; ^5^ State Key Laboratory of Pharmaceutical Biotechnology The University of Hong Kong Hong Kong SAR China

**Keywords:** aortic arch calcification, essential metals, mediation effect, mixed exposure

## Abstract

Vascular calcification is a strong predictor of cardiovascular events. Essential metals play critical roles in maintaining human health. However, the association of essential metal levels with risk of aortic arch calcification (AoAC) remains unclear. We measured the plasma concentrations of nine essential metals in a cross‐sectional population and evaluated their individual and combined effects on AoAC risk using multiple statistical methods. We also explored the mediating role of fasting glucose. In the logistic regression model, higher quartiles of magnesium and copper were associated with the decreased AoAC risk, while higher quartile of manganese was associated with higher AoAC risk. The least absolute shrinkage and selection operator penalized regression analysis identified magnesium, manganese, calcium, cobalt, and copper as key metals associated with AoAC risk. The weighted quantile sum regression suggested a combined effect of metal mixture. A linear and positive dose–response relationship was found between manganese and AoAC in males. Moreover, blood glucose might mediate a proportion of 9.38% of the association between manganese exposure and AoAC risk. In summary, five essential metal levels were associated with AoAC and showed combined effect. Fasting glucose might play a significant role in mediating manganese exposure‐associated AoAC risk.

## Graphical abstract

This study aimed to evaluate the relationships between essential metal levels and aortic arch calcification (AoAC) risk in a mid‐aged and older population of Shenzhen, China. The results showed that plasma levels of five essential metals were associated with AoAC and showed combined effect. Blood glucose played a significant role in mediating Mn exposure‐associated AoAC risks.

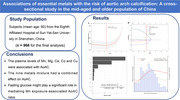

## 1 INTRODUCTION

Cardiovascular diseases (CVDs) remained the leading cause of health damage worldwide.^1^ According to the Global Burden of Disease Study 2019, the prevalence of CVD in China increased from 4.8% to 5.8% during 1990−2019, and it still ranked the first cause of death in China.^2^ Traditional risk factors of CVD include age, gender, dyslipidemia, obesity, hypertension, chronic kidney disease (CKD), diabetes mellitus, and so on.^3^ Vascular calcification (VC), mainly characterized by aberrant calcium (Ca) phosphate crystal deposition in the vessel wall, is a common pathological manifestation of atherosclerosis, hypertension, CKD, and aging.^4^, ^5^ Other mechanisms including cell senescence, inflammation, and epigenetic regulation were also implicated.^6^, ^7^ Aortic arch calcification (AoAC), a common and easily recognized type of VC, was usually accompanied with advanced CKD and might contribute to increased risk of major cardiac adverse events^8^, ^9^ and the mortality of CKD.^10^ Because of the heavy burden to human health and social economy, it is critical to identify novel risk factors for the development of AoAC and take interventions for prevention.

Essential metals might perform a critical role in the development of VC.^11^ Essential metal ions function as protein cofactors in various biological processes, and exert beneficial effects on human health. For example, previous studies have shown that the trace elements, such as copper (Cu) and manganese (Mn), acted as metal cofactors and were essential for normal activity of superoxide dismutases, which were the principal antioxidant defense mechanisms against (O₂•‐) in terms of atherosclerosis, hypertension and angiogenesis.^12^ Magnesium (Mg) could inhibit the osteogenic differentiation and bone gene expression of vascular smooth muscle cells (VSMCs) and led to reduced VC.^13^ Zinc (Zn) inhibited hypoxia‐inducible factor prolyl hydroxylase inhibitors which could aggravate calcification of VSMCs caused by high phosphate.^14^ However, it could also be toxic if the concentrations did not lie in the appropriate range.^15^ A cross‐sectional study indicated that higher plasma concentration of Mn could increase the risk of hypertension by 1.57‐fold in the Korean population.^16^ In addition, higher levels of Mg and Ca were potentially related to the development of CVDs, including coronary heart disease, hypertension and arrhythmias.^17^ However, human studies reporting associations between essential metal levels and AoAC risk were extremely rare, and the limited studies only focused on single or few metals.^18^, ^19^ Therefore, the associations between levels of multiple essential metals and the risk of AoAC are largely unknown and warrant investigations considering the serious hazard of AoAC on human cardiovascular health.

Metals are also closely related with glucose homeostasis. A cross‐sectional study, conducted in a general population (sample size: 696) in Shimen of China, observed a positive and joint effect of five metals on the fasting blood glucose (FBG) levels.^20^ Consistently, a prospective American Indian family‐based cohort study revealed a positive linkage between Arsenic exposure and FBG concentration in 1047 participants.^21^ Existing evidence showed that triglyceride (TG)–glucose index, which was linked to insulin resistance, contributed to carotid atherosclerosis^22^ and calcification of abdominal aorta.^23^ A cross‐sectional study conducted in 1419 US participants also confirmed the positive association between FBG and abdominal aortic calcification.^22^ Therefore, we supposed that the glucose homeostasis might play a mediating role relating metal exposure and AoAC risk.

In this study, we performed a cross‐sectional study in a mid‐aged and older population of Shenzhen, China and the plasma levels of nine essential metals, including Mg, Mn, selenium (Se), Cu, cobalt (Co), Zn, (iron) Fe, molybdenum (Mo), and Ca were detected. Multiple advanced statistical methods such as least absolute shrinkage and selection operator (LASSO) penalized regression analysis, weighted quantile sum regression (WQS) and restricted cubic spline (RCS) models were employed to identify the individual and combined effect of these essential metals on AoAC risk.

## 2 RESULTS

### 2.1 The general characteristics and plasma metal concentrations of the study participants

Table 1 showed the general characteristics and the plasma metal concentrations of the study participants. More men were found in the AoAC group (*p *< 0.001). It was found that subjects with AoAC were older (*p *< 0.001) and had higher SBP levels (*p *< 0.001). Meanwhile, diabetes and hypertension were more prevalent in the AoAC group than in the non‐AoAC group (*p *< 0.05). As for the metal levels, the median plasma concentrations of the nine essential metals varied from 0.24 µg/L to 80276.95 µg/L (Table S1). The AoAC group had higher Mo concentration (*p* = 0.01) and lower Co concentration (*p* = 0.002) than the non‐AoAC group. In terms of concentrations of other metals, there was no significant difference between the two groups (all *p* > 0.05). Spearman correlation coefficients (*r*_s_) between the metals varied from −0.08 to 0.55 (Figure S1). According to the results of the analysis of the covariance (ANCOVA), with the degrees of AoAC increased, the levels of plasma Mo tended to increase (*p*‐trend < 0.05), while the trend of plasma Co was the opposite (*p*‐trend < 0.05; Figure S2).

**TABLE 1:** General characteristics and plasma metal concentrations of the study participants.

Variables	Total population (*n* = 966)	Nonaortic arch calcification (*n* = 542)	Aortic arch calcification (*n* = 424)	*p* Value
Age, years	60 (54, 67)	58 (51, 63)	63 (59, 70)	<0.001
Male, *n* (%)	563 (58.28)	283 (52.21)	280 (66.04)	<0.001
BMI (kg/m^2^)	23.62 (21.87, 25.61)	23.66 (21.91, 25.65)	23.53 (21.74, 25.39)	0.244
SBP (mmHg)	130 (120, 138)	126 (120, 136)	131 (123, 139)	<0.001
DBP (mmHg)	80 (76, 86)	80 (76, 87)	80 (75, 85)	0.263
Smoking, *n* (%)	123 (12.73)	79 (14.58)	44 (10.38)	0.065
Drinking, *n* (%)	110 (11.39)	71 (13.1)	39 (9.2)	0.073
TG (mmol/L)	1.28 (0.88, 1.84)	1.23 (0.87, 1.85)	1.30 (0.90, 1.83)	0.274
TC (mmol/L)	5.38 (4.67, 6.11)	5.39 (4.63, 6.10)	5.38 (4.69, 6.13)	0.495
HDL‐c (mmol/L)	1.40 (1.17, 1.62)	1.39 (1.15, 1.61)	1.40 (1.17, 1.63)	0.352
LDL‐c (mmol/L)	3.07 (2.50, 3.70)	3.10 (2.54, 3.68)	2.98 (2.49, 3.71)	0.196
eGFR (mL/min/1.73 m^2^)	98.98 (85.23, 113.85)	99.97 (85.98, 115.21)	97.8 (83.60, 112.00)	0.085
UA, µmol/L	333 (283, 396)	331 (285, 392)	334 (282, 402)	0.515
Hypertension, *n* (%)	304 (31.47)	137 (25.28)	167 (39.39)	<0.001
Diabetes, *n* (%)	105 (10.87)	47 (8.67)	58 (13.68)	0.017
Hyperlipidemia, *n* (%)	103 (10.66)	51 (9.41)	52 (12.26)	0.186
**Metal concentration (µg/L)**				
Magnesium	20571.16 (18881.87, 22449.36)	20596.1 (18981.65, 22393.34)	20525.21 (18781.32, 22501.59)	0.586
Manganese	1.01 (0.62, 1.56)	0.98 (0.59, 1.47)	1.03 (0.65, 1.7)	0.126
Calcium	80276.95 (75096.02, 88874.26)	80167.29 (75390.35, 89035.91)	80404.92 (74614.41, 88809.48)	0.736
Iron	1697.04 (1314.38, 2264.9)	1730.03 (1320.54, 2261.24)	1672.25 (1313.45, 2283.09)	0.642
Cobalt	0.24 (0.19, 0.29)	0.24 (0.2, 0.29)	0.23 (0.17, 0.28)	0.002
Copper	945.74 (807.73, 1075.12)	939.15 (801.13, 1059.84)	956.16 (810.78, 1085.93)	0.184
Zinc	1052.32 (903.3, 1253.77)	1045.72 (906.28, 1235.86)	1065.05 (902, 1266.86)	0.404
Selenium	100.22 (88.11, 115.9)	100.17 (87.21, 115.87)	100.6 (89.24, 115.84)	0.517
Molybdenum	1.09 (0.88, 1.36)	1.06 (0.86, 1.31)	1.11 (0.91, 1.42)	0.010

*Note*: Data were presented as numbers (percentages) for categorical data or median (interquartile range) for nonparametrically distributed data. We used Wilcoxon rank sum test to compare the continuous variables based on the data distribution and the Chi‐square test to compare the categorical variables.

Abbreviations: BMI, body mass index; DBP, diastolic blood pressure; eGFR, nephron glomerular filtration rate; HDL‐c, high‐density lipoprotein cholesterol; LDL‐c, low‐density lipoprotein cholesterol; SBP, systolic blood pressure; TC, total cholesterol; TG, triglyceride; UA, uric acid.

### 2.2 Associations of plasma essential metals with AoAC

The relationships between the essential metals and AoAC were shown in Table 2 by the logistic regression models. After adjusting for age, gender, body mass index (BMI), smoking and alcohol drinking (model 1), compared with the first quartile, the ORs (95% CIs) were 0.66 (0.44, 0.97) for Cu and 0.66 (0.45, 0.97) for Mg in the third quartile, and 1.63 (1.11, 2.39) for Mn in the fourth quartile. After additional adjusting for hypertension and diabetes (model 2), the ORs were 0.67 (0.45, 0.99) for Mg and 0.65 (0.44, 0.97) for Cu in the third quartile, and 1.64 (1.12, 2.41) for Mn in the highest quartile, respectively. The results of the model 3 were similar to those of the model 2, even after adjusting for total cholesterol (TC), TG, estimated glomerular filtration rate (eGFR) and uric acid (UA). The ORs were 0.66 (0.44, 0.97) for Mg and 0.65 (0.44, 0.97) for Cu in the third quartile, and 1.66 (1.13, 2.44) for Mn in the highest quartile. Moreover, the plasma levels of Mn showed an increased trend with AoAC risk (*p*‐trend < 0.05). In summary, the results revealed that Cu, Mg, and Mn were significantly associated with AoAC risk.

**TABLE 2:** Odds ratios (95%CI) for the AoAC risk correlated with quartiles of plasma metals according to the logistic regression analysis.

	Metal concentrations (µg/L)				
Metal	Q1	Q2	Q3	Q4	*p*‐Trend
Mg	<18881.87	18881.87–20571.16	20571.16–22449.36	>22449.36	
*n* (controls/cases)	242/113	241/100	241/101	242/110	
Crude	1.00(Ref)	0.81 (0.56,1.16)	0.82 (0.57,1.18)	0.95 (0.67,1.36)	0.785
Model 1	1.00(Ref)	0.74 (0.50,1.09)	0.66 (0.45,0.97)	0.77 (0.53,1.14)	0.152
Model 2	1.00(Ref)	0.73 (0.50,1.08)	0.67 (0.45,0.99)	0.79 (0.54,1.17)	0.199
Model 3	1.00(Ref)	0.72 (0.49,1.06)	0.66 (0.44,0.97)	0.78 (0.53,1.15)	0.175
Mn	<0.62	0.62–1.01	1.01–1.56	>1.56	
*n* (controls/cases)	242/96	241/106	241/102	242/120	
Crude	1.00(Ref)	1.19 (0.83,1.71)	1.12 (0.78,1.60)	1.50 (1.04,2.14)	0.043
Model 1	1.00(Ref)	1.63 (1.10,2.41)	1.48 (1.00,2.19)	1.63 (1.11,2.39)	0.021
Model 2	1.00(Ref)	1.64 (1.10,2.43)	1.49 (1.00,2.20)	1.64 (1.12,2.41)	0.020
Model 3	1.00(Ref)	1.62 (1.09,2.40)	1.48 (1.00,2.20)	1.66 (1.13,2.44)	0.018
Ca	<75,096.02	75,096.02–80,276.95	80,276.95–88,874.26	>88,874.26	
*n* (controls/cases)	242/114	241/93	241/112	242/105	
Crude	1.00(Ref)	0.71 (0.49,1.01)	0.97 (0.68,1.39)	0.86 (0.60,1.23)	0.744
Model 1	1.00(Ref)	0.83 (0.57,1.23)	1.38 (0.94,2.03)	1.29 (0.87,1.91)	0.070
Model 2	1.00(Ref)	0.84 (0.57,1.24)	1.38 (0.94,2.04)	1.30 (0.87,1.93)	0.069
Model 3	1.00(Ref)	0.84 (0.57,1.23)	1.38 (0.94,2.05)	1.30 (0.88,1.94)	0.067
Fe	<1314.38	1314.38–1697.04	1697.04–2264.90	>2264.90	
*n* (controls/cases)	241/107	241/112	241/97	241/107	
Crude	1.00(Ref)	1.09 (0.76,1.56)	0.84 (0.59,1.21)	1.00 (0.70,1.43)	0.693
Model 1	1.00(Ref)	1.23 (0.84,1.81)	1.09 (0.73,1.62)	1.41 (0.94,2.12)	0.156
Model 2	1.00(Ref)	1.25 (0.85,1.84)	1.11 (0.74,1.65)	1.44 (0.96,2.16)	0.136
Model 3	1.00(Ref)	1.24 (0.84,1.84)	1.12 (0.75,1.67)	1.44 (0.96,2.17)	0.130
Co	<0.19	0.19–0.24	0.24–0.29	>0.29	
*n* (controls/cases)	237/122	237/105	237/95	237/96	
Crude	1.00(Ref)	0.75 (0.52,1.08)	0.63 (0.44,0.91)	0.64 (0.45,0.92)	0.007
Model 1	1.00(Ref)	0.81 (0.56,1.19)	0.78 (0.53,1.15)	0.92 (0.62,1.36)	0.454
Model 2	1.00(Ref)	0.80 (0.55,1.18)	0.77 (0.52,1.14)	0.92 (0.62,1.36)	0.445
Model 3	1.00(Ref)	0.80 (0.54,1.18)	0.77 (0.52,1.14)	0.93 (0.63,1.38)	0.481
Cu	<807.73	807.73–945.74	945.74–1075.12	>1075.12	
*n* (controls/cases)	242/104	241/99	241/104	242/117	
Crude	1.00(Ref)	0.93 (0.64,1.33)	1.01 (0.70,1.44)	1.24 (0.87,1.78)	0.215
Model 1	1.00(Ref)	0.71 (0.48,1.05)	0.66 (0.44,0.97)	0.76 (0.51,1.14)	0.175
Model 2	1.00(Ref)	0.70 (0.47,1.03)	0.65 (0.44,0.97)	0.77 (0.51,1.15)	0.185
Model 3	1.00(Ref)	0.70 (0.47,1.04)	0.65 (0.44,0.97)	0.76 (0.51,1.14)	0.183
Zn	<903.3	903.30–1052.32	1052.32–1253.77	>1253.77	
*n* (controls/cases)	241/107	241/97	240/105	240/113	
Crude	1.00(Ref)	0.84 (0.59,1.21)	0.97 (0.68,1.40)	1.11 (0.77,1.58)	0.469
Model 1	1.00(Ref)	0.76 (0.51,1.12)	0.83 (0.57,1.23)	0.93 (0.63,1.37)	0.826
Model 2	1.00(Ref)	0.75 (0.51,1.11)	0.83 (0.56,1.23)	0.92 (0.62,1.36)	0.805
Model 3	1.00(Ref)	0.73 (0.49,1.08)	0.81 (0.55,1.20)	0.89 (0.6,1.32)	0.696
Se	<88.11	88.11–100.22	100.22–115.90	>115.90	
*n* (controls/cases)	242/98	241/112	241/108	242/106	
Crude	1.00(Ref)	1.28 (0.89,1.83)	1.19 (0.83,1.71)	1.15 (0.80,1.64)	0.554
Model 1	1.00(Ref)	1.25 (0.85,1.83)	1.16 (0.79,1.71)	1.08 (0.73,1.59)	0.803
Model 2	1.00(Ref)	1.23 (0.83,1.81)	1.14 (0.77,1.68)	1.02 (0.69,1.52)	0.997
Model 3	1.00(Ref)	1.23 (0.83,1.81)	1.12 (0.76,1.65)	1.01 (0.68,1.50)	0.933
Mo	<0.88	0.88–1.09	1.09–1.36	>1.36	
*n* (controls/cases)	242/94	242/105	241/105	242/120	
Crude	1.00(Ref)	1.22 (0.85,1.75)	1.22 (0.85,1.75)	1.55 (1.08,2.22)	0.022
Model 1	1.00(Ref)	1.01 (0.69,1.49)	0.82 (0.55,1.22)	0.98 (0.66,1.46)	0.721
Model 2	1.00(Ref)	1.02 (0.69,1.50)	0.82 (0.55,1.22)	0.98 (0.65,1.46)	0.710
Model 3	1.00(Ref)	1.03 (0.70,1.52)	0.83 (0.55,1.23)	0.97 (0.65,1.46)	0.702

*Note*: We obtained the *p* values for trend test from the logistic regression analysis by using the median of each metal quartile as a continuous variable. Model 1 was controlled for gender, age, BMI, alcohol drinking, and smoking; model 2 was further controlled for hypertension and diabetes; model 3 was controlled for TG, TC, eGFR, and UA based on model 2.

Abbreviations: AoAC, aortic arch calcification; Ca, calcium; Co, cobalt; Cu, copper; eGFR, estimated glomerular filtration rate; Fe, iron; Mg, magnesium; Mn, manganese; Mo, molybdenum; Se, selenium; TG, triglycerides; TC, total cholesterol; UA, uric acid; Zn, zinc.

### 2.3 Selection of independent metals associated with the risk of AoAC

**FIGURE 1:**
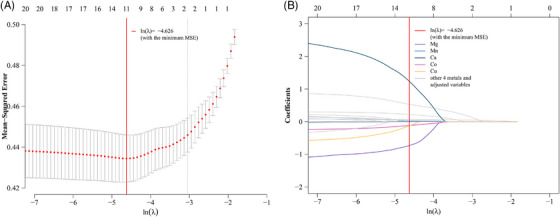
The LASSO penalized regression analysis for the relationships between multiple plasma metals and AoAC risk. The analysis used AoAC as the outcome variable and nine ln‐transformed plasma concentrations of metals as the predictor variables, with adjustment for gender, BMI, age, alcohol drinking and smoking, diabetes, hypertension, TG, TC, eGFR, and UA. The cross‐validation curve along the λ sequence (Figure 1A) was represented by the black dotted line with its upper and lower standard deviation curves (error bars). Coefficient profiles for plasma metals were displayed by Figure 1B. AoAC, aortic arch calcification; BMI, body mass index; TC, total cholesterol; TG, triglycerides; eGFR, estimated glomerular filtration rate; UA, uric acid.

The LASSO penalized regression was further applied to screen out the key metals related to the risk of AoAC after repeating 1000 times (Figure S3). Mg, Mn, Ca, Co, and Cu were identified within the optimal ln *λ* (−4.63). Among them, Ca and Mn were identified to be positively associated with AoAC, while Mg, Cu, and Co were found to be negatively associated with AoAC (Figure 1).

### 2.4 Combined effect of multiple metals

**FIGURE 2:**
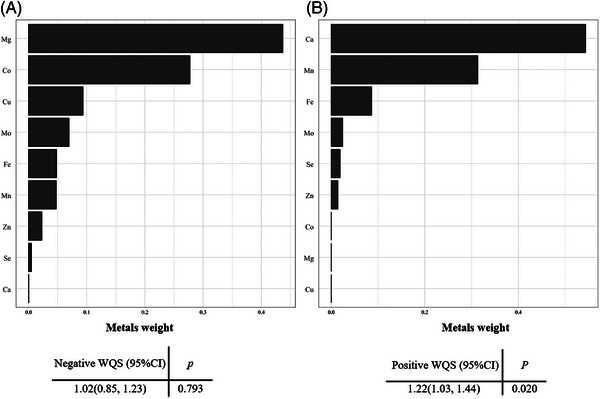
WQS model regression index weights for AoAC risk in the total population. Each metal was assigned weights of the WQS regression model in positive direction (B) and negative direction (A). The analysis was controlled for gender, age, BMI, alcohol drinking and smoking, diabetes, hypertension, TG, TC, eGFR and UA. AoAC, aortic arch calcification; WQS regression, weighted quantile sum regression; BMI, body mass index; TC, total cholesterol; TG, triglycerides; eGFR, estimated glomerular filtration rate; UA, uric acid.

The WQS regression model with all metals included was applied to explore the combined effect of the metal mixture. The findings indicated that the WQS index was positively related to increased AoAC risk (OR:1.22, 95% CI: 1.03–1.44) after confounders adjustment. The most significant metals positively associated with risk of AoAC were Ca (weighted 0.54) and Mn (weighted 0.31). In contrast, the relationship between the metal mixture and AoAC in the total population was not significant in the negative direction (Figure 2).

### 2.5 Dose–response relationships between plasma metals and AoAC risk

**FIGURE 3:**
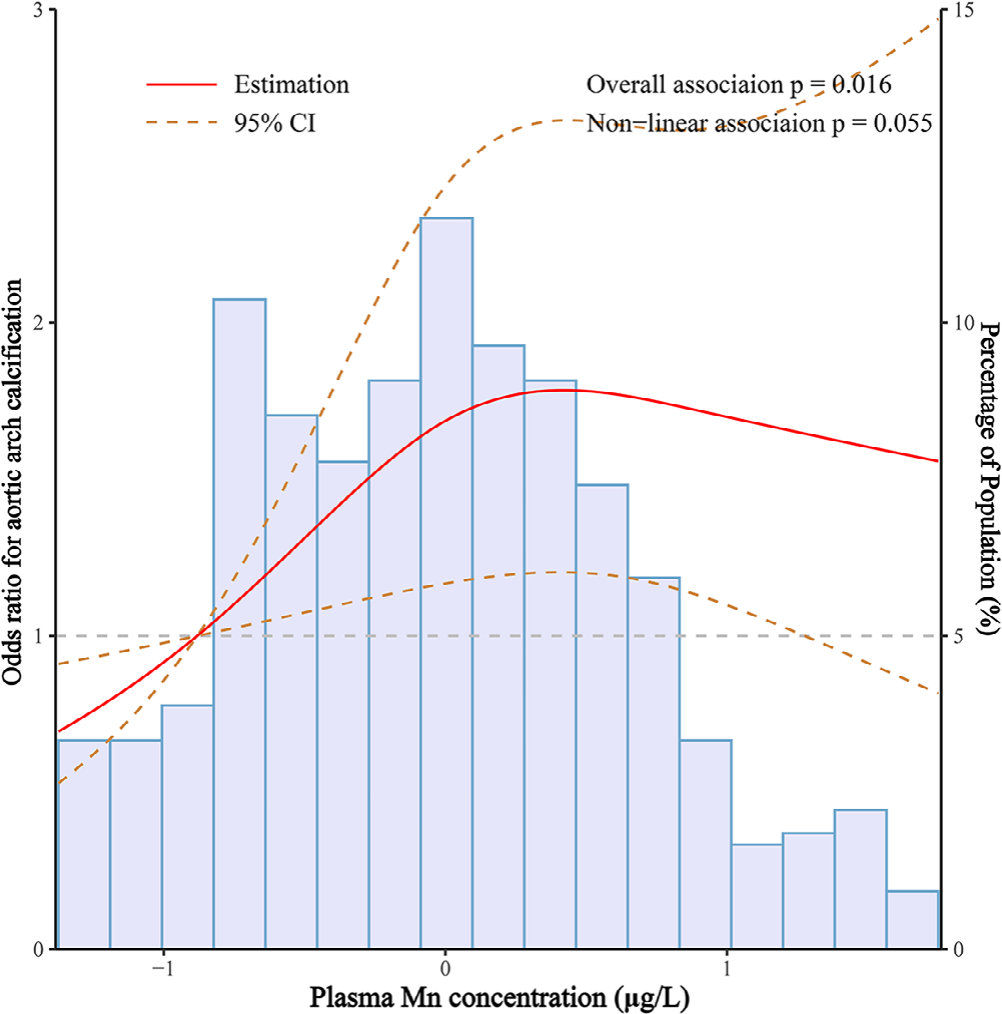
The RCS model for the relationships between plasma Mn concentration and AoAC risk in males. The RCS models for the ln‐transformed concentrations of plasma Mn showed the 95% confidence intervals (long dashed lines) and adjusted odds ratios (solid red lines). We set the reference values at the 10th percentiles, and the knots at the 90th, 50^th^, and 10th percentiles of the ln‐transformed concentrations, respectively. Controlled confounding factors were in line with the logistic regression Model 3. RCS, restricted cubic spline; AoAC, aortic arch calcification; Mn, Manganese.

A dose–response relationships between plasma essential metals and AoAC risk was further investigated using the RCS regression. Since five metals (Mg, Mn, Ca, Co, Cu) in total were identified through the LASSO model and the logistic regression analysis, we included these metals in the dose–response relationship analysis. There were no significant dose–response relationships between five plasma metals and AoAC risk in the whole population (Figure S4), while in males, a linear and positive association was found between Mn levels and risk of AoAC (*p* for nonlinearity = 0.055, *p* for overall association = 0.016; Figure 3). No significant dose–response association was found between plasma metals and the risk of AoAC in females or other plasma metals and AoAC risks in males (all *p* for overall association and *p* for nonlinear association > 0.05; Figures S5 and S6).

### 2.6 Subgroup analysis

To identify the susceptible population, the above mentioned five metals were dichotomized as high or low levels based on the median of the non‐AoAC group. In comparison with the low level of Ca, the ORs (95% CI) were 1.60 (1.12, 2.31), 1.42 (1.05, 1.91), 1.60 (1.06, 2.43), 1.57 (1.12, 2.22), and 15.62 (1.75, 331.33) for men, nondrinkers, overweight or obese people, people without hypertension, and people with CKD, respectively. There were positive associations between the high level of Mn and AoAC risk in males and people with diabetes, and the ORs (95% CI) were 1.51 (1.05, 2.17) and 3.02 (1.22, 7.97), respectively. However, negative associations between the high level of metals (Cu, Co) and AoAC risk were found in people with diabetes, youngers and females, respectively. A significant interaction was detected between dichotomized Mn and gender (*p*‐interaction = 0.045). Meanwhile, there were interaction effects among dichotomized Co with both age and hyperlipidemia (*p*‐interaction = 0.042 and 0.036, respectively). Results were demonstrated in Figures S7–S11.

### 2.7 Mediating role of blood glucose on the association between plasma Mn and AoAC risk

Mediation model was employed to evaluate the proportion of the association between plasma Mn levels and AoAC risk that could be explained by mediating factors, as showed in Table 3. The results indicated that blood glucose showed a significant mediation effect on the relationship between plasma Mn levels and AoAC risk in males (the proportion mediated ratios obtained was 9.38%). However, no significant mediation effect was found in the whole population or in the females (Tables S2 and S3).

**TABLE 3:** Assessment of mediating effects by GLU on the association between plasma Mn level and AoAC risk in males.

	Direct effect	Indirect effect	Total effect	Proportion of mediation
GLU	1.05 (0.99, 1.11)	1.01 (1.00, 1.01)	1.06 (1.01,1.12)	9.38%

*Note*: Model was adjusted for BMI, age, alcohol drinking and smoking status, diabetes, hypertension, TG, TC, eGFR, and UA.

Abbreviations: AoAC, aortic arch calcification; GLU, blood glucose; Mn, manganese.

## 3 DISCUSSION

In this cross‐sectional study, we explored the relationships between nine plasma essential metals and AoAC risk by employing multi‐pollutant statistical models. Logistic regression model revealed that Mn or Cu/Mg was positively or negatively associated with AoAC. Five essential metals including Mg, Mn, Cu, Co, and Ca were identified to be the key metals influencing the development of AoAC by LASSO, and their combined effect was validated by the WQS model. Furthermore, the plasma level of Mn contributed to the AoAC risk in a positive and linear dose–response manner in males. Moreover, we revealed that blood glucose might partially mediate the relationship between Mn and AoAC risk.

Dietary Mn is essential for proper immune function, reproduction, regulating blood sugar and cellular energy, bone growth, digestion, hemostasis, blood coagulation, and fighting reactive oxygen species.^24^ Mn mainly enters human body through the dietary sources, whole grains, legumes, nuts, and tea contain the highest amounts of Mn, while a minor fraction was gained from dermal intake and inhalation.^25^ Due to its abundant dietary sources, Mg deficiency is extremely unusual. However, Mg could also be hazard to human if its concentration exceeds the threshold. Mn toxicity is mostly attributable to the environmental exposures such as water drinking and airborne exposure.^24^ Mn overload can cause progressive, permanent and neurodegenerative damage, resulting in syndromes similar to idiopathic Parkinson's disease.^26^ A study among 3200 welders in Beijing, China found that the concentrations of serum Mn in welders diagnosed with Mn poisoning ranged from 3 to 36 µg/L and were significantly higher than control subjects.^27^ According to our research, the median concentration of plasma Mn was 1.01 µg/L in the whole population, while a slightly higher median blood Mn concentration of 1.97 µg/L was revealed in the North Chinese population (Hebei Province).^28^ The different concentrations of metals may attribute to differences in age, dietary intake, genetic variation, and region.^29^ In our study, plasma Mn levels were positively and linearly related to AoAC risk, with subjects in the highest quartile of Mn (>1.56 g/L) at a 1.64‐fold greater risk of AoAC than those in the lowest quartile (≤0.62 µg/L). Although there was limited evidence showing the relationship between Mn and AoAC, Mn plays a significant role in CVD. For instance, the results from the EPIC‐Potsdam cohort study revealed that a higher concentration of Mn (>1.04 µg/L) was related to increased risk of CVD (OR: 1.15, 95% CI: 1.02−1.30).^30^ In addition, a cross‐sectional study of the Korea National Health and Nutrition Examination Survey indicated that high plasma Mn (>1.40 µg/dL) increased the risk of hypertension by 1.567‐fold.^16^ On the contrary, the National Health and Nutrition Examination Survey (NHANES) data (2011−2014) from a cross‐sectional survey showed that higher urinary Mn levels (>0.18 µg/L) were negatively related to both diastolic and systolic blood pressure.^31^ To the best of our knowledge, this is the first human study focusing on the relationship between internal exposure level of Mn and the occurrence of AoAC, and these findings need to be validated in further large cohort study. Animal studies have reported that excess Mn could be toxic to cardiac muscle cells and tissues. Infusions of Mn dipyridoxyl diphosphate or Mn chloride resulted in increased coronary vascular resistance and aortic pressure in isolated perfused hearts.^32^ In addition, an in vitro study aiming to explore toxic components or emission sources of PM2.5 demonstrated that Mn significantly induced the accumulation of reactive oxygen species levels in VSMCs.^33^ VC was defined as an active process partly driven by VSMCs trans‐differentiation within the vascular wall.^10^ However, the evidence about whether or how chronic Mn exposure may cause AoAC remains sparse.

In recent years, a growing number of studies have proved the relationship between Mn levels and blood glucose. For instance, a cross‐sectional study based on coke oven workers showed an association between urinary Mn levels and hyperglycemia risk.^34^ Besides, plasma glucose concentrations in rats were significantly enhanced by subchronic injections of MnO_2_ microparticles and nanoparticles.^35^ Also, recent studies have found that high glucose concentrations could accelerate the calcification of HVSMCs.^36^, ^37^ According to the above findings, the mediation analysis was further performed in our research. We observed a significant mediated effect of blood glucose on the relationship between Mn and AoAC risk in males, among which the mediated proportion of blood glucose was 9.38%. Furthermore, the subgroup analysis demonstrated that the males and diabetics groups were more susceptible to AoAC risk due to elevated plasma Mn levels. Likewise, a previous study showed that compared with female population, males suffered from a twofold increased risk of developing cardiovascular calcification.^38^ We also found that the linear and positive association between Mn and AoAC was only found in males. The gender difference in the correlation between metals and AoAC may be attributed to several factors, such as the differences in the environmental exposure levels and hormonal regulation between different gender. Males and females may have different sources and routes of metal exposure, such as occupational, dietary, and environmental factors.^39^, ^40^ These differences may result in different plasma concentrations and dietary intakes of essential metals in males and females. Another possible factor that may cause the gender difference is the different effects of sex hormones on metal metabolism and VC. Sex hormones, including estrogen and testosterone, can affect the levels and functions of some metals, such as Cu, Mn, and Zn, in the human body. These metals can modulate the synthesis, transport, and signaling of sex hormones in return. For instance, estrogen may inhibit the expression of divalent metal transporter 1, a protein that facilitates the uptake of Mn and other metals.^41^ Moreover, sex hormones may influence the susceptibility of vascular tissues to calcification by affecting the expression of calcification inhibitors and promoters. For example, estrogen may protect against VC by enhancing the expression of matrix Gla protein, a potent inhibitor of calcification.^42^ Testosterone, on the other hand, may promote VC by increasing the expression of osteopontin, a procalcific protein.^38^

Mg, the fourth most rich mineral in human body, can be obtained from green leafy vegetables, whole grains, nuts, and legumes. It was recognized as a cofactor for more than 300 enzymatic reactions, and was important for adenosine triphosphate metabolism.^43^ The half‐life of blood Mg was 4 h.^44^ In our research, the result of logistic regression and the LASSO model both revealed that Mg was negatively related to AoAC risk. Our findings were consistent with previous reports. For example, a single center cross‐sectional study consisted of 80 prevalent peritoneal dialysis patients suggested that a higher serum Mg level (>0.8 mmol/L) was related to a lower abdominal aortic calcification score (*β* = −7.81, *p* = 0.03).^45^ Besides, a randomized clinical trial (RCT) enrolling 125 subjects showed that oral Mg oxide supplement retarded the progression of coronary artery calcification in patients with CKD.^46^ However, another RCT revealed that Mg supplementation for 12 months was not effective in slowing the VC progression in CKD.^47^ More studies are needed to explore the optimal dose, duration, and timing of Mg supplementation in the prevention or treatment of VC in CKD. The in vitro study also discovered that Mg suppressed the calcification of VSMCs by inhibiting their osteoblastic transdifferentiation, apoptosis, and Wnt/*β*‐catenin signaling.^13^ However, the molecular mechanisms underlying the anticalcification effects of Mg are still not fully elucidated.

Cu, a trace element, is critical for enzyme function, and exerts biological functions as both prooxidant and antioxidant.^17^ The main dietary sources of Cu are foods rich in protein, such as organ meats, shellfish, crustacean species, nuts, seeds, as well as whole grains and chocolate.^48^ According to our research, the median concentration of plasma Cu in the whole population was 945.74 µg/L, which was lower than the data obtained from 150 middle‐aged Taiwanese adults (1007.89 µg/L).^49^ The effect of Cu level on the cardiovascular system was controversial. Within a normal biological range, the beneficial influence of Cu on the cardiovascular system and overall health has been extensively studied.^50^ Consistently, we identified Cu to be negatively associated with VC at the dose between 945.74 and 1075.12 µg/L. However, a cross‐sectional study consisted of 6754 subjects from rural China indicated that high plasma concentration of Cu (>1190 µg/L) was positively correlated with hypertension risk.^51^ Likewise, researchers found that high serum Cu level (>1110 µg/L) was associated with increased risks of atherosclerotic CVD in a cohort study of 2492 middle‐aged Finnish men.^52^ However, similar result was not found in a cross‐sectional study of the NHANES 2007−2014 in US adults.^53^ The Multi‐Ethnic Study of Atherosclerosis (MESA) found that higher urinary Cu levels were associated with the progression of coronary artery calcification in 6418 participants over a 10‐year period.^54^ The inconsistent results of the studies might be attributed to deficiency or excess of Cu about the study population. Cu deficiency could lead to a reduction in cardiac metabolism and energy supply, and produces various risk factors associated with ischemic heart disease.^55^ Conversely, excessive Cu could induce oxidative stress, forming a Cu–homocysteine complex possibly leading to vascular damage and endothelial dysfunction.^56^ In vivo animal experiment also demonstrated that prolonged excessive Cu intake could result in impaired cardiac function and myocardial injury, which might attribute to the activation of mitochondria‐dependent apoptotic signaling.^57^ Recent studies have also highlighted the role of Cu homeostasis and cuproptosis, a novel form of regulated cell death, in the development and progression of CVDs.^58^ VC is a complex cellular and molecular process, involving the osteochondrogenic transformation of VSMCs, the disturbance of Ca‐phosphate metabolism, inflammation and oxidative stress.^4^ Abnormal levels of Cu may cause oxidative stress and inflammatory responses, which may accelerate the progression of VC, but the specific mechanisms are not clear. More studies are needed to explore the causal relationship and potential therapeutic targets of Cu in the development of VC.

Co is an important component of vitamin B12, which is abundant in shellfish, organ meats, seaweed, and fermented soy products.^59^ In our study, the median level of plasma Co in the whole population was 0.24 µg/L, while a lower median blood Co concentration of 0.194 µg/L was reported among the general population in China.^60^ A cross‐sectional study conducted in 3389 participants from the NHANES survey (2015−2016) of America revealed that elevated serum Co levels were positively correlated with a higher CVD prevalence in a nonlinear manner, indicating that Co exposure could be a risk factor for CVD.^61^ In our study, the LASSO model revealed that Co was negatively related to the AoAC risk. In MESA, higher urinary Co levels might contribute to coronary artery calcification, which was different from the result in our study.^54^ However, the relationship between blood Co level and the CAC risk was not found in a study conducted in the hemodialysis patients.^62^ An animal experiment demonstrated that high‐dose Co ion concentrations in serum might have toxic effects on heart which showed obvious hyperemia and swelling of the heart.^63^ In addition, the exposure of 300‐ and 600‐ppm concentrations of Co chloride relative to the control could cause hypertension and cardiovascular complications in rats, in which the pathologic mechanisms might be involved in oxidative stress, inflammation, and apoptosis.^64^

Ca is the 5th most rich element in our body. Approximately 99% of Ca is contained in the skeleton in the form of hydroxyapatite, which is a complex Ca phosphate molecule.^65^ In addition to supporting bone health, Ca plays a vital role in nerve transmission, muscle contractions and blood clotting. However, excess Ca can also deposit in the soft tissues, especially the arteries, and then cause calcification. In our research, Ca was screened out positively related to AoAC risk by the WQS and LASSO models, suggesting that higher plasma Ca levels might increase the likelihood of AoAC. Several studies have found that Ca was involved in the initiation and progression of VC. Ca has the ability to bind to phosphate and facilitate the formation of Ca phosphate crystals, serving as nucleation sites for subsequent calcification. Additionally, Ca can stimulate the expression of osteogenic genes and promote the differentiation of VSMCs into osteoblast‐like cells, leading to the secretion of matrix vesicles and mineralization of the extracellular matrix. Moreover, previous studies have proved that Ca could modulate the activity of calcification inhibitors, such as matrix Gla protein and fetuin‐A, and reduce their protective effects on the vascular wall.^66^ A RCT based on 425 subjects demonstrated that reducing dialysate Ca concentrations could slow down coronary artery calcification progression and promote bone turnover in patients with hemodialysis while under treatment with ≥1.50 mmol/L Ca dialysate.^67^ A population study suggested that Ca might be a critical mediator of VSMCs damage and calcification in CKD patients, while no similar effect was found on vessels in healthy control subjects.^68^ However, there was limited evidence about association between plasma Ca level and VC in general population. Therefore, more researches are needed to clarify the role of Ca in VC and the potential mechanisms involved.

Our research offers several advantages. First, this is the first study to explore the associations between exposure to multiple essential metals and AoAC in China. In addition, we applied the WQS model, which could handle the complex correlation patterns among multiple variables, to evaluate the combined effect of multiple metals on AoAC. Furthermore, we explored and identified the possible mechanism through which fasting glucose mediated the association between metal exposure and AoAC risk, based on the evidence that glucose homeostasis was involved in VC and could be influenced by metal exposure. However, several limitations should also be addressed. First, the reverse causation could not be prevented based on the cross‐sectional study design, and the causal relationship between metal exposure and AoAC needs to be replicated in prospective cohort studies. Besides, due to insufficient manpower and materials, our study was conducted in a single center with a relatively small sample size, which may limit the generalizability of our findings to the whole population of China, Further studies with larger and more representative samples are needed to confirm our results. We also acknowledge that our study did not account for the influence of diet and genetic predisposition on metal levels, which might bias the association between metal exposure and AoAC. The study population in our study belongs to Shenzhen, China, where people have a high consumption of seafood. Seafood is a major source of dietary exposure to metals, especially Mn, Cu, Mg, and Ca.^69^ Therefore, the regional diet habit of Shenzhen may be both related with plasma metal levels and the risk of AoAC, and further investigations should include diet as the potential confounding factor. Another factor that may influence the plasma metal levels is genetic predisposition. Genetic variations in metal transporters, detoxification enzymes, and mineral metabolism regulators may affect the absorption, distribution, excretion, and toxicity of metals in the human body.^70^ For instance, polymorphisms in fibroblast growth factor 23 (FGF23), a hormone that regulates phosphate and Ca homeostasis, have been related to VC in CKD patients^71^ Modification effects on the relationship of Mn levels in urine with the risk of dyslexia in children were suggested by rs27072 in the *SLC6A3* gene, which may reduce the affinity of the dopamine transporter for Mn, increase the intracellular accumulation of Mn, and thus ultimately modulate its neurotoxic effects. In summary, it is plausible that genetic predisposition plays a role in modulating the plasma metal levels and their effects on AoAC.^72^ Fourth, one spot plasma sample might cause exposure misclassification, and the repeated measurements are needed in the future study. Finally, we used the plasma levels as internal exposure biomarkers for all metals, which might not be optimal for some metals and cause the misclassificaiton of exposure levels.

## 4 MATERIALS AND METHODS

### 4.1 Study population and design

This is a cross‐sectional study consisted of 966 subjects who participated in routine physical examinations at the Eighth Affiliated Hospital of Sun Yat‐Sen University (Shenzhen, China) from 2015 to 2017. The flowchart of population enrollment was shown in Figure S12. The subjects with complete demographic data, medical history, results of blood biochemical examination and essential metals examination were included, and the participants with neurological diseases, cerebrovascular disease, malignancies, peripheral arterial occlusive disease, or without chest X‐ray examination were excluded. A total of 966 participants were ultimately enrolled in this research, among whom 424 were subjects with AoAC. Our research was approved by the ethics committees of the Shenzhen Center for Disease Control and Prevention. All subjects gave their written informed consent.

### 4.2 Measurement of covariates

A professional questionnaire was applied to gather the general characteristics, lifestyles, and medical histories of the study population by skilled healthcare staff. The basic information of the questionnaire included gender, age, alcohol drinking, smoking, diabetes history, hyperlipidemia history, and hypertension history. The definition of diabetes included a FBG level of ≥ 7.0 mmol/L, self‐reported diabetes diagnosis, or taking hypoglycemic medicine. Hypertension was defined as having a systolic blood pressure of at least 140 mmHg or a diastolic blood pressure of at least 90 mmHg for at least two measurements, or those who have been diagnosed with hypertension and taking antihypertensive medicine.^73^ Individuals who consumed alcohol on a weekly basis for a period exceeding 6 months were considered alcohol drinkers. Subjects who smoked daily and lasted for over 6 months were defined smokers, including former and current smokers. The glucose, low‐density lipoprotein cholesterol, high‐density lipoprotein cholesterol, TG, TC, UA, creatinine, and other indexes were detected in fasting blood samples by hospital. After the centrifugation of venous blood samples, the plasma samples were stored at −80° until laboratory analysis.

### 4.3 Detection of plasma metals concentration

We detected plasma concentrations of nine essential metals, including Ca, Mn, Fe, Mg, Co, Cu, Zn, Se, and Mo by the inductively coupled plasma mass spectrometry (ICP‐MS, Agilent 7700 series; Agilent Technologies, USA), and the details of detection method were described in the Supplementary Methods and in the previous studies.^74^, ^75^ In a word, the blood samples were acidified with 300 µL of 55% (v/v) HNO3 (TAMAPURE‐AA 10 ultrapure analytical reagent; Tamachemicals CO., Kawasaki, Japan) for 2 h at room temperature and then heated in a boiling water bath until they turned pale yellow. The samples were cooled and diluted with ultrapure water to 6.0 mL for further analysis. Ultimately, fewer than 10% of sample values were below the detection limits (LODs). Table S1 shows the intra‐ and interassay coefficients and the LODs of the plasma metals. LOD/√2 was used to replace the values of plasma metal concentration that were lower than the LOD.

### 4.4 AoAC index calculation

Detection of the presence and severity of AoAC were obtained by plain chest X‐ray examination. The chest radiograph was taken while the subject was standing in the rear forward position and holding his breath with deep inspiration. AoAC was calculated by two experienced radiologists who were blinded to the participant's general information. As previously demonstrated,^76^ each plain chest radiograph was divided into 16 sections based on the aortic arch and specified a calcification index noted as a percentage graded from 0 to 2, of which grade 0, 1, 2 was non‐AoAC, 0 < AoAC score < 50%, and AoAC score ≥ 50%, respectively.

### 4.5 Statistical analysis

The study population's general characteristics were presented using percentages or medians with interquartile ranges. Statistical tests such as the Wilcoxon rank sum test or Chi‐square test were employed to assess differences in general characteristics between the AoAC and non‐AoAC groups. Spearman correlation analysis was conducted to explore associations between metals, while the ANCOVA model was utilized to examine the relationship between plasma metal concentrations and varying degrees of AoAC.

In order to investigate the relationship between essential metals and the risk of AoAC, a logistic regression model was utilized to calculate ORs and 95% CIs. The study participants were categorized into quartiles based on the distribution of metal concentrations in the non‐AoAC group, with the first quartile serving as the reference value. Model 1 was adjusted for gender, age, BMI, alcohol drinking, and smoking status; model 2 included additional adjustments for diabetes and hypertension on top of model 1; model 3 accounted for TG, TC, eGFR, and UA in addition to the variables in model 2. The median value of each metal quartile was treated as a continuous variable in the analysis.

The LASSO penalized regression analysis is essentially a coefficient compression method that shrinks the coefficients of nonsignificantly influential variables to zero which could realize both predictor selection and parameter estimation at the same time.^77^, ^78^ The greater the *λ*, the more the penalty for the linear model with more variables, and eventually a model with fewer variables is achieved. In the context of that, we conducted LASSO penalized regression analysis with all metals entered in the model to select the crucial metals for AoAC by 10‐fold cross‐validation with penalty term lambda. The adjusted covariates were consistent with those in model 3 of the logistic regression analysis.

Additionally, WQS regression was employed to assess the combined effect of all the elements on AoAC. The detailed description of WQS regression has been published in a previous study.^79^ In the WQS model, all metals were treated as a whole, and we supposed it exerted effect on the risk of AoAC in the same direction, negative or positive. The WQS index, calculated from the accumulation of the product of each metal weight (ranged from 0 to 1 and added up 1) and the scored quartiles of individual metals could represent the total body burden of all nine essential metals. In order to maintain the stability of the final results, we split the sample size into a training set (50%) and a validation set (50%) with 1000 bootstrap samples.

Moreover, the RCS analysis (with three knots: 10th, 50th, and 90th percentiles) was employed to analyze the dose–response relationships between the essential metals and AoAC risk. The ln‐transformed metal concentration at the 10th percentiles were set as the reference value. R package “mediation” was employed to evaluate the potential mediating effect of FPG on the associations of essential metals with AoAC risk.

Subgroup analysis was also conducted. The stratification variables consisted of age (≤ 60, > 60), gender, BMI (< 24, ≥ 24), alcohol drinking (yes, no), smoking (yes, no), diabetes (yes, no), hypertension (yes, no), hyperlipidemia (yes, no), and CKD (yes, no). The definition of CKD was described as eGFR < 90 mL/min/1.73 m^2^ combined with an albumin–creatinine ratio ≥ 30 mg/g, based on the KDIGO 2012 for CKD diagnosis.

All the analyses involved in this study were analyzed with R software (4.0.2; Lucent Technologies, USA) and IBM SPSS 25.0 (SPSS, Chicago, IL), and a two‐sided *p* < 0.05 indicated statistical significance.

## 5 CONCLUSIONS

Our study confirmed that Mn, Mg, Ca, Co, and Cu in plasma were associated with AoAC risk, and fasting glucose partially mediated the positive and linear dose–response relationship between Mn and AoAC in males. Furthermore, the mixture‐chemical analysis indicated the positive correlation between plasma metal coexposure and AoAC risk. Our findings may suggest important public health implications in prevention of AoAC and provide scientific evidence for the establishment of environmental and nutritional health standard in China. Moreover, this study also had important clinical implications. We provided valuable clues for the clinical doctors who can improve the management of VC by taking into consideration of plasma metals detection at diagnosis and an adequate nutritional supplement advice with essential elements, bringing health benefits in preventing AoAC and even other serious CVDs. Further cohort studies and experimental studies are required to validate our findings and to explore the underlying mechanism.

## AUTHOR CONTRIBUTIONS

*Conceptualization, writing review and editing, and writing—original draft*: Mingxing Mo. *Data collection, writing review and editing, and writing—original draft*: Li Yin; *Software, formal analysis, and data curation*: Tian Wang; *Resources, writing review and editing*: Ziquan Lv. *Validation and investigation*: Yadi Guo. *Conceptualization, writing review and editing*: Jiangang Shen and Huanji Zhang. *Formal analysis and data curation*: Ning Liu and Qiuling Wang. *Funding acquisition, resources, and supervision*: Suli Huang. *Visualization, project administration, writing review and editing, and funding acquisition*: Hui Huang. All authors have read and approved the final manuscript.

## CONFLICT OF INTEREST STATEMENT

The authors declare that they have no conflict of interest.

## ETHICS STATEMENT

This study was conducted in accordance with the Declaration of Helsinki and was approved by the Ethics Committee of Shenzhen Center for Disease Control and Prevention (approval number: QS2019030016). All participants provided written informed consent before enrollment.

## Supporting information

Supporting Information

## Data Availability

Data will be available from the corresponding author upon reasonable request.
