# Supplementary material for: Associations of essential metals with the risk of aortic arch calcification: a cross‐sectional study in a mid‐aged and older population of Shenzhen, China
Source: MedComm (2020). 2024 May 13;5(5):e533. doi: 10.1002/mco2.533 (PMC11091022; doi:10.1002/mco2.533)
Supplement: Supplementary file 1 — Supporting Information [file MCO2-5-e533-s001.docx]

**Supplementary material**

**Associations of essential metals with the risk of aortic arch calcification: A cross-sectional study** **in** **a mid-aged and older population of Shenzhen, China**

Mingxing Mo ^1, #^, Li Yin ^1, #^, Tian Wang ^3, #^, Ziquan Lv ^3^, Yadi Guo ^1^, Jiangang Shen^4, 5^, Huanji Zhang ^1^, Ning Liu ^3^, Qiuling Wang ^3^, Suli Huang ^2, 3,^ *, Hui Huang ^1,^ *

^1^ Department of Cardiology, Joint Laboratory of Guangdong-Hong Kong-Macao Universities for Nutritional Metabolism and Precise Prevention and Control of Major Chronic Diseases, the Eighth Affiliated Hospital, Sun Yat-sen University, Shenzhen, 518033, China

^2^ School of Public Health, Shenzhen University Medical School, Shenzhen University, Shenzhen, Guangdong, 518055, China

^3^ Shenzhen Center for Disease control and Prevention, Shenzhen, 518055, China

^4^ School of Chinese Medicine, Li Ka Shing Faculty of Medicine, The University of Hong Kong, Hong Kong SAR 999077, China

^5^ State Key Laboratory of Pharmaceutical Biotechnology, The University of Hong Kong, Hong Kong SAR 999077, China.

^#^ These authors contributed equally to this work.

* Corresponding authors at: School of Public Health, Shenzhen University Medical School, Shenzhen University, Shenzhen, Guangdong, 518055, China; Shenzhen Center for Disease control and Prevention, Shenzhen, 518055, China (S. Huang); Department of Cardiology, Joint Laboratory of Guangdong-Hong Kong-Macao Universities for Nutritional Metabolism and Precise Prevention and Control of Major Chronic Diseases, the Eighth Affiliated Hospital, Sun Yat-sen University, Shenzhen, 518033, China (H. Huang)

E-mail addresses: [huangsuli420@163.com](mailto:huangsuli420@163.com) (S. Huang); [huangh8@mail.sysu.edu.cn](mailto:huangh8@mail.sysu.edu.cn) (H. Huang)

**Appendix S1: Plasma metal concentration detection**

We applied the inductively coupled plasma mass spectrometry (ICP-MS, Agilent 7700 series, Agilent Technologies, USA) to detect the plasma metals as previously reported ^1^.

The wet acid digestion method was used to digest blood samples. To begin with, we incubate plasma sample with 300 μL 55% (v/v) HNO3 (TAMAPURE-AA 10 ultrapure analytical reagent, Tamachemicals CO., Kawasaki, Japan) at room temperature for 2 h, then the samples were transferred to the water bath at boiling temperature until the solution turned light yellow. After cooling, the sample was diluted to 6.0 mL with ultrapure water for final analysis. The certified reference materials (Clin Chek human plasma controls for trace elements no. 8883 and no. 8884, Recipe, Munich, Germany) was applied to evaluate the accuracy and precision of the detection. The measured values of the reference materials were within the recommended ranges for each metal. Besides, standard reference materials 1640a (Trace Elements in Natural Water, National Institute of Standards and Technology, Gaithersburg, MD, USA) were analyzed to assess the instrument performance every 30 samples.

**Reference**

1. Wen Y, Huang S, Zhang Y, et al. Associations of multiple plasma metals with the risk of ischemic stroke: A case-control study. *Environ Int.* 2019;125:125-134.

**Table of Contents**

**Table S1.** Limits of detection, distributions of plasma metals among the study population, intra-assay and inter-assay coefficients of variation (n=966).

**Table S2.** Assessment of mediating effects by GLU on the association between plasma Mn level and AoAC risk in the whole population.

**Table S3.** Assessment of mediating effects by GLU on the association between plasma Mn level and AoAC risk in females.

**Figure S1.** Correlations between plasma metals among the study population (n=966).

**Figure S2.** Associations between the concentration of plasma metals and degrees of AoAC.

**Figure S3.** The result of variables after repeating 1000 times in LASSO penalized regression analysis.

**Figure S4.** The restricted cubic spline for the associations between plasma metal concentrations and the risk of AoAC in the whole population.

**Figure S5.** The RCS model for the relationships between plasma Mg, Ca, Co, Cu concentrations and AoAC risks in males.

**Figure S6.** The restricted cubic spline for the associations between plasma metal concentrations and the risk of AoAC in females.

**Figure S7**. Subgroup analysis of the association between Mn and AoAC.

**Figure S8.** Subgroup analysis of the association between Mg and AoAC.

**Figure S9**. Subgroup analysis of the association between Cu and AoAC.

**Figure S10**. Subgroup analysis of the association between Ca and AoAC.

**Figure S11**. Subgroup analysis of the association between Co and AoAC.

**Figure S12.** Flowchart of population enrollment in this study.

| **Table S1. Limits of detection, distributions of plasma metals among the study population, intra-assay and inter-assay coefficients of variation (n=966)** | | | | | | | | | | | |
| --- | --- | --- | --- | --- | --- | --- | --- | --- | --- | --- | --- |
| **Plasma metals (μg/L)** | **LOD** | **Total No. (%) <LOD** a | **non-Aortic arch calcification <LOD** a | **Aortic arch calcification <LOD** a | **5th percentile** | **25th percentile** | **50th percentile** | **75th percentile** | **95th percentile** | **Intra-assay CV%** | **Inter-assay CV%** |
| Magnesium | 100.00 | 0 (0.00) | 0 (0.00) | 0 (0.00) | 16490.99 | 18881.87 | 20571.16 | 22449.36 | 25010.25 | 0.05 | 0.08 |
| Manganese | 0.20 | 40 (4.14) | 24 (4.43) | 16 (3.77) | 0.22 | 0.62 | 1.01 | 1.56 | 3.27 | 7.20 | 8.97 |
| Calcium | 1000.00 | 0 (0.00) | 0 (0.00) | 0 (0.00) | 67253.37 | 75096.02 | 80276.95 | 88874.26 | 104003.96 | 0.06 | 0.08 |
| Iron | 100.00 | 0 (0.00) | 0 (0.00) | 0 (0.00) | 894.36 | 1314.38 | 1697.04 | 2264.90 | 3488.70 | 5.92 | 7.47 |
| Cobalt | 0.01 | 1 (0.11) | 1 (0.24) | 0 (0.00) | 0.07 | 0.19 | 0.24 | 0.29 | 0.40 | 4.39 | 5.87 |
| Copper | 5.00 | 0 (0.00) | 0 (0.00) | 0 (0.00) | 641.19 | 807.73 | 945.74 | 1075.12 | 1323.96 | 3.76 | 4.39 |
| Zinc | 300.00 | 0 (0.00) | 0 (0.00) | 0 (0.00) | 726.38 | 903.30 | 1052.32 | 1253.77 | 1576.93 | 3.77 | 4.08 |
| Selenium | 0.30 | 0 (0.00) | 0 (0.00) | 0 (0.00) | 71.64 | 88.11 | 100.22 | 115.90 | 141.36 | 3.48 | 4.54 |
| Molybdenum | 0.10 | 0 (0.00) | 0 (0.00) | 0 (0.00) | 0.64 | 0.88 | 1.09 | 1.36 | 1.96 | 1.41 | 6.48 |

Note: LOD, the limit of detection; CV, coefficient of variance. Data are presented as numbers (percentages).

| **Table S2. Assessment of mediating effects by GLU on the association between plasma Mn level and AoAC risk in the whole population.** | | | | |
| --- | --- | --- | --- | --- |
|  | Direct effect | Indirect effect | Total effect | Proportion of mediation |
| GLU | 1.03(0.99, 1.07) | 1.00 (0.99, 1.01) | 1.04 (0.99,1.07) | 6.46% |

Note: Model was adjusted for age, BMI, sex, smoking and alcohol drinking status, hypertension, diabetes, TG, TC, eGFR and UA. GLU, blood glucose; Mn, Manganese; AoAC, aortic arch calcification; BMI, body mass index; TC, total cholesterol; TG, triglycerides; eGFR, estimated glomerular filtration rate; UA, uric acid.

| **Table S3. Assessment of mediating effects by GLU on the association between plasma Mn level and AoAC risk in females.** | | | | |
| --- | --- | --- | --- | --- |
|  | Direct effect | Indirect effect | Total effect | Proportion of mediation |
| GLU | 1.01(0.94, 1.07) | 0.99(0.98, 1.01) | 0.99(0.93, 1.07) | 0.18% |

Note: Model was adjusted for age, BMI, smoking and alcohol drinking status, hypertension, diabetes, TG, TC, eGFR and UA. GLU, blood glucose; Mn, Manganese; AoAC, aortic arch calcification; BMI, body mass index; TC, total cholesterol; TG, triglycerides; eGFR, estimated glomerular filtration rate; UA, uric acid.


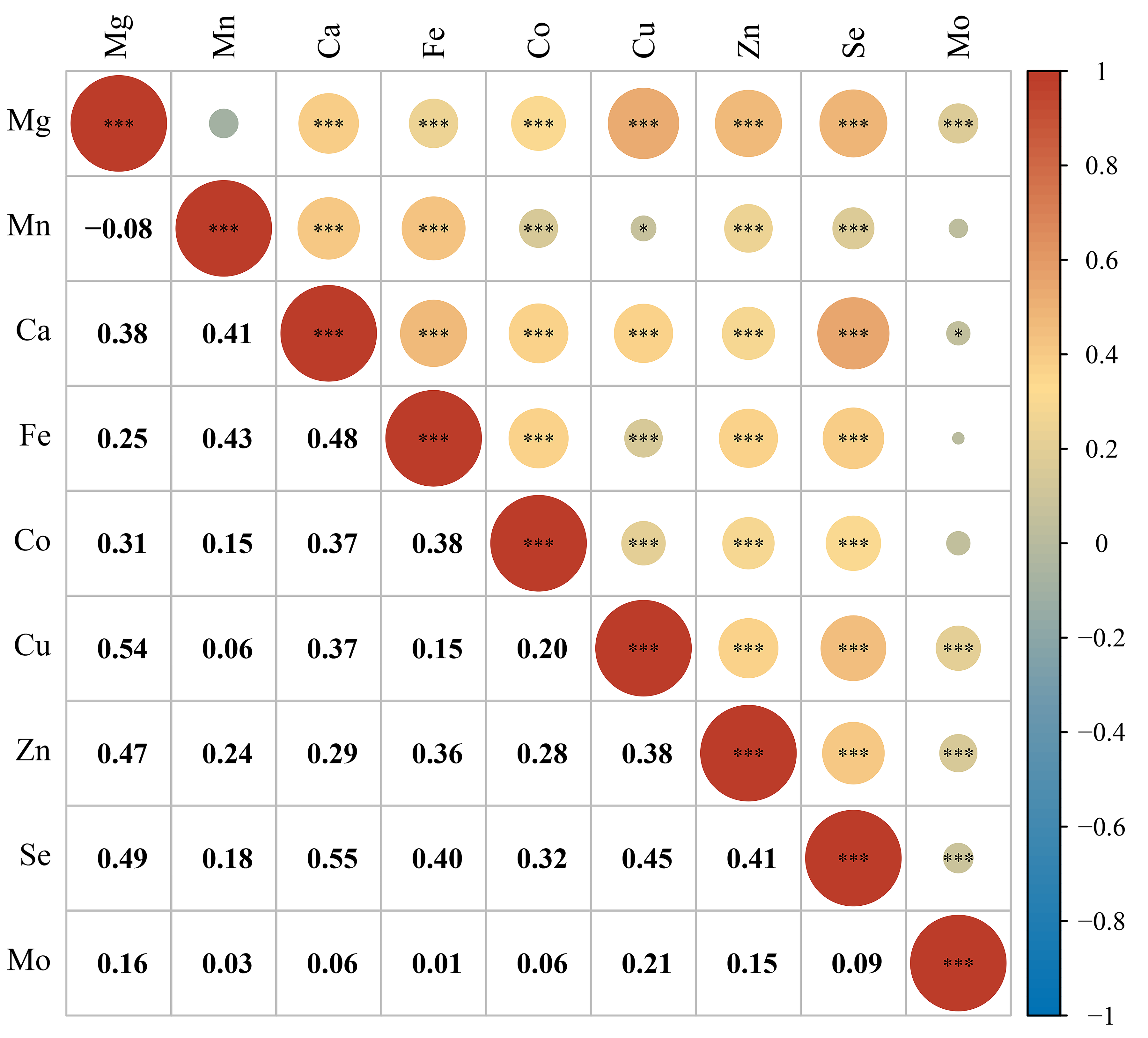


**Figure S1. Correlations between plasma metals among the study population (n=966).**

Note: Spearman correlation coefficients are presented. The colors and areas of circles reflect the values of corresponding correlation coefficients. Mg, Magnesium; Mn, Manganese; Ca, Calcium; Fe, Iron; Co, Cobalt; Cu, Copper; Zn, Zinc; Se, Selenium; Mo, Molybdenum. * *p* < 0.05, ***p* < 0.01, *** *p* < 0.001


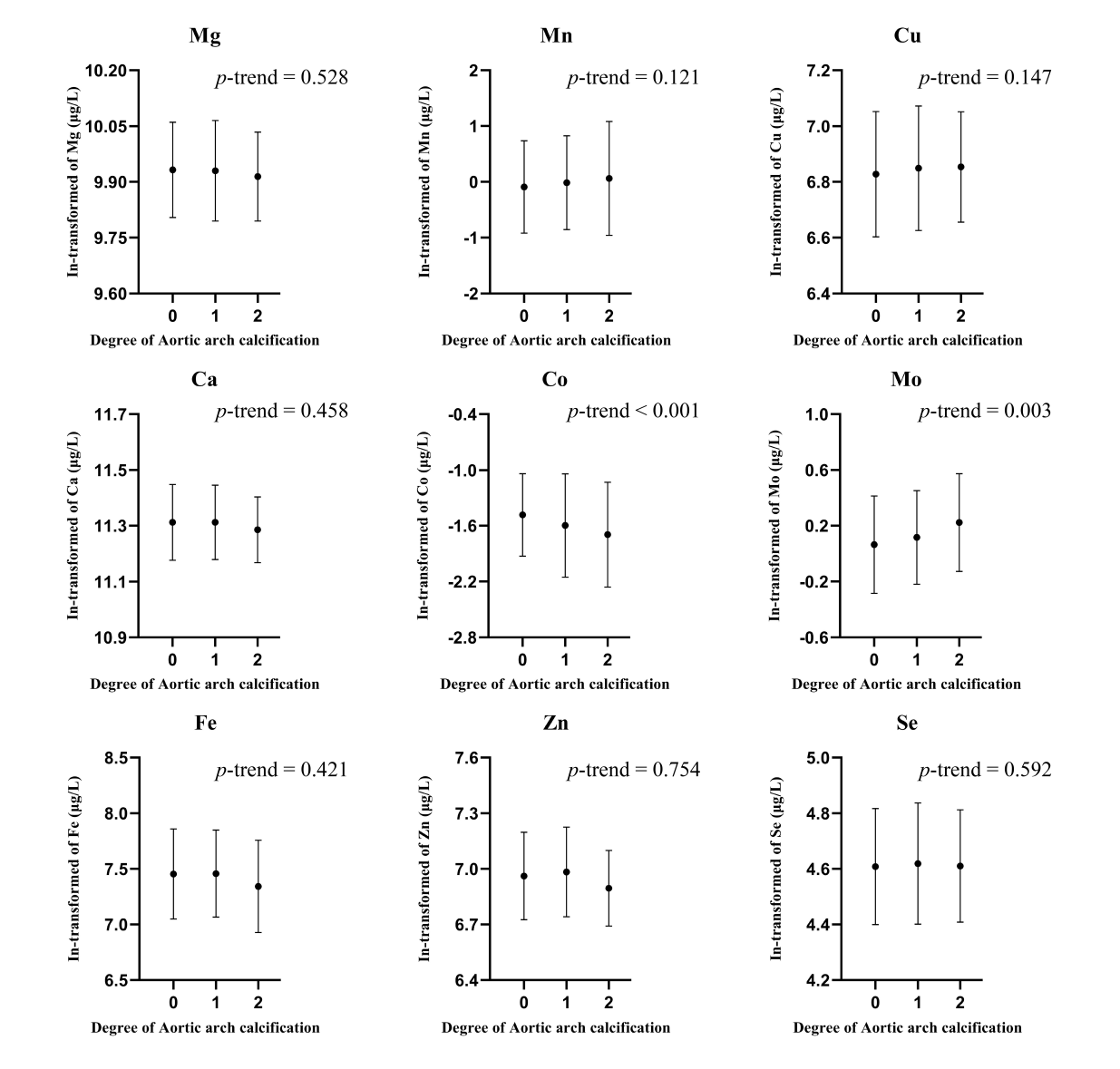


**Figure S2. Associations between the concentration of plasma metals and degrees of AoAC.**

Note: The analysis of covariance (ANCOVA) model included the ln-transformed concentration of plasma metal Mg, Mn, Cu, Ca, Co, Mo, Fe, Zn and Se as the dependent variables, and degrees of aortic arch calcification as a fixed factor, adjusted for gender, age, BMI, alcohol drinking and smoking status, diabetes, hypertension, TG, TC, eGFR and UA. Degrees of aortic arch calcification: 0 (non-AAC); 1 (moderate calcification): 0＜AoAC score＜50%; 2 (severe calcification): AoAC score ≥ 50%. Mg, Magnesium; Mn, Manganese; Ca, Calcium; Fe, Iron; Co, Cobalt; Cu, Copper; Zn, Zinc; Se, Selenium; Mo, Molybdenum. AoAC, aortic arch calcification; BMI, body mass index; TC, total cholesterol; TG, triglycerides; eGFR, estimated glomerular filtration rate; UA, uric acid.


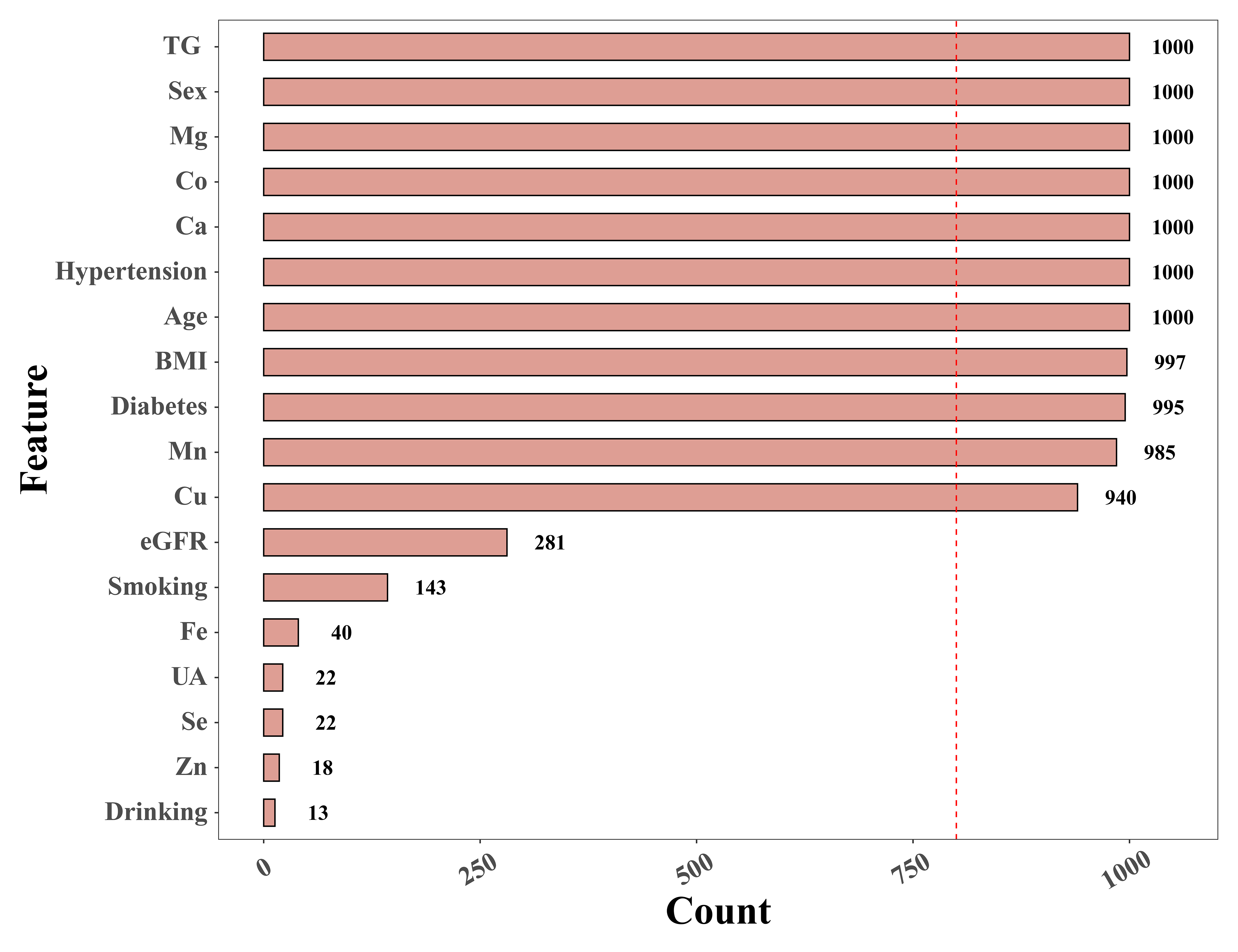


**Figure S3. The result of variables after repeating 1000 times in LASSO** **penalized regression analysis.**

Note: BMI, body mass index; TG, triglyceride; eGFR, nephron glomerular filtration rate; UA, uric acid. Mg, Magnesium; Mn, Manganese; Ca, Calcium; Fe, Iron; Co, Cobalt; Cu, Copper; Zn, Zinc; Se, Selenium; BMI, body mass index; TG, triglycerides; eGFR, estimated glomerular filtration rate; UA, uric acid.


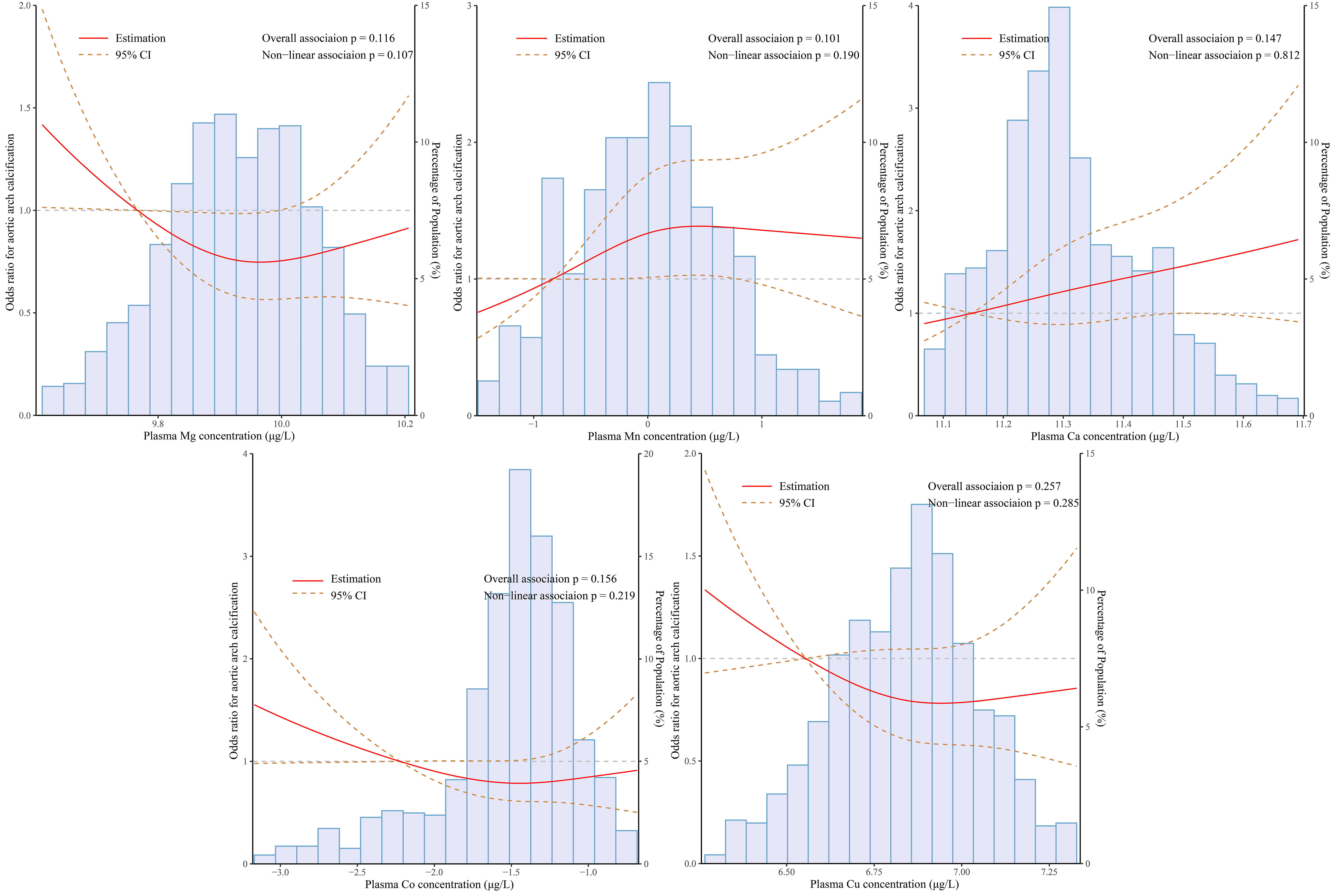


**Figure S4. The restricted cubic spline for the associations between plasma metal concentrations and the risk of AoAC in the whole population.**

Note: The lines represent adjusted odds ratios (solid red lines) and 95% confidence intervals (long dashed lines) based on the restricted cubic spline models for the ln-transformed concentrations of plasma Mg, Mn, Co, Ca and Cu. The reference values were set at the 10th percentiles, and the knots were set at the 10th, 50th and 90th percentiles of the ln-transformed concentrations, respectively. Adjusted factors were consistent with model 3 of the logistic regression analysis. AoAC, aortic arch calcification; Mg, magnesium; Mn, Manganese; Ca, calcium; Co, cobalt; Cu, copper.


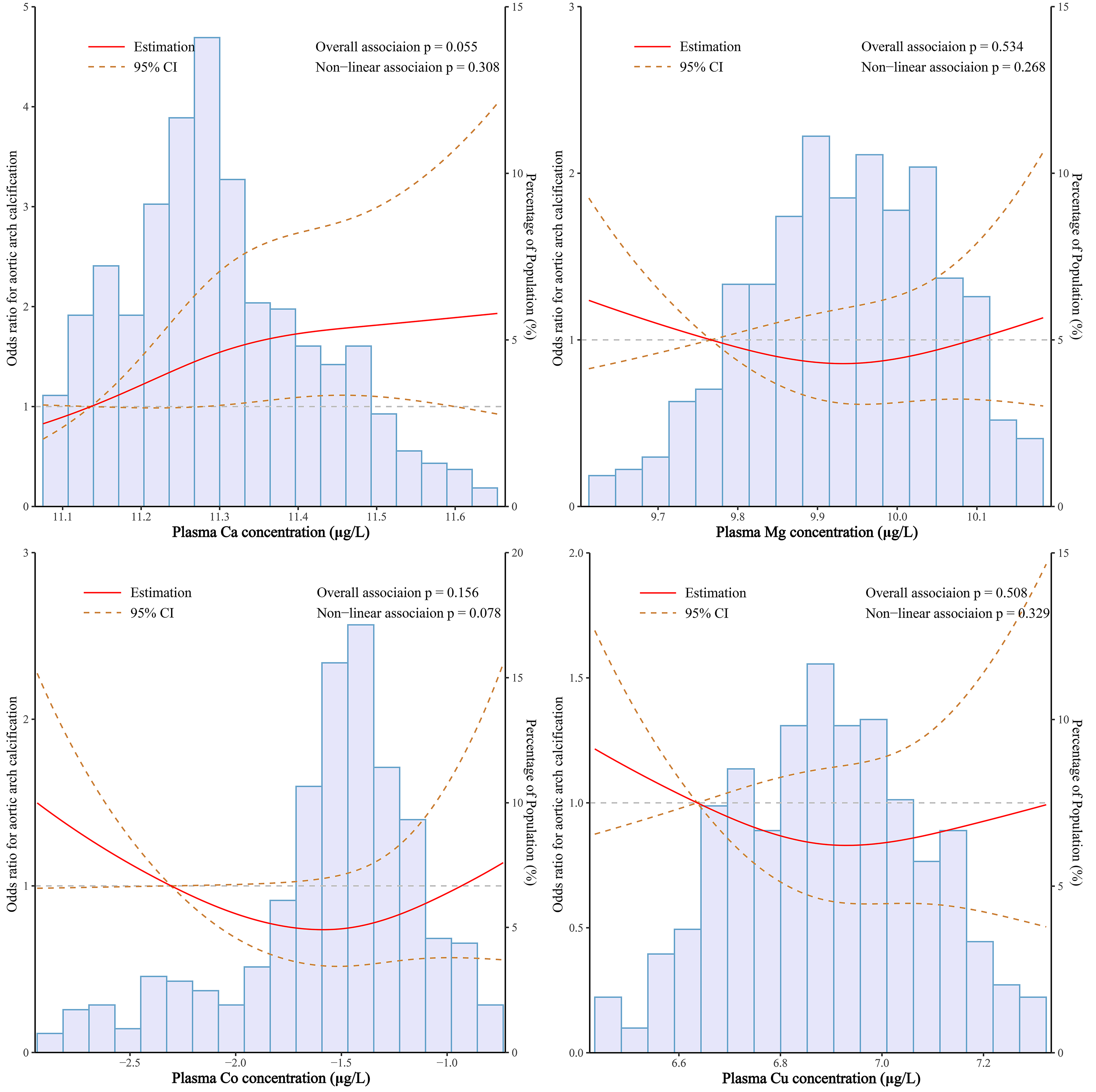


**Figure S5. The restricted cubic spline for the relationships between plasma Mg, Ca, Co, Cu concentrations and AoAC risks in males.**

Note: The restricted cubic spline models for the ln-transformed concentrations of plasma Mg, Ca, Co and Cu showed the 95% confidence intervals (long dashed lines) and adjusted odds ratios (solid red lines). We set the reference values at the 10th percentiles, and the knots at the 90th, 50th and 10th percentiles of the ln-transformed concentrations, respectively. Controlled confounding factors were in line with the logistic regression Model 3. AoAC, aortic arch calcification; Mg, Magnesium; Ca, Calcium; Co, Cobalt; Cu, Copper.


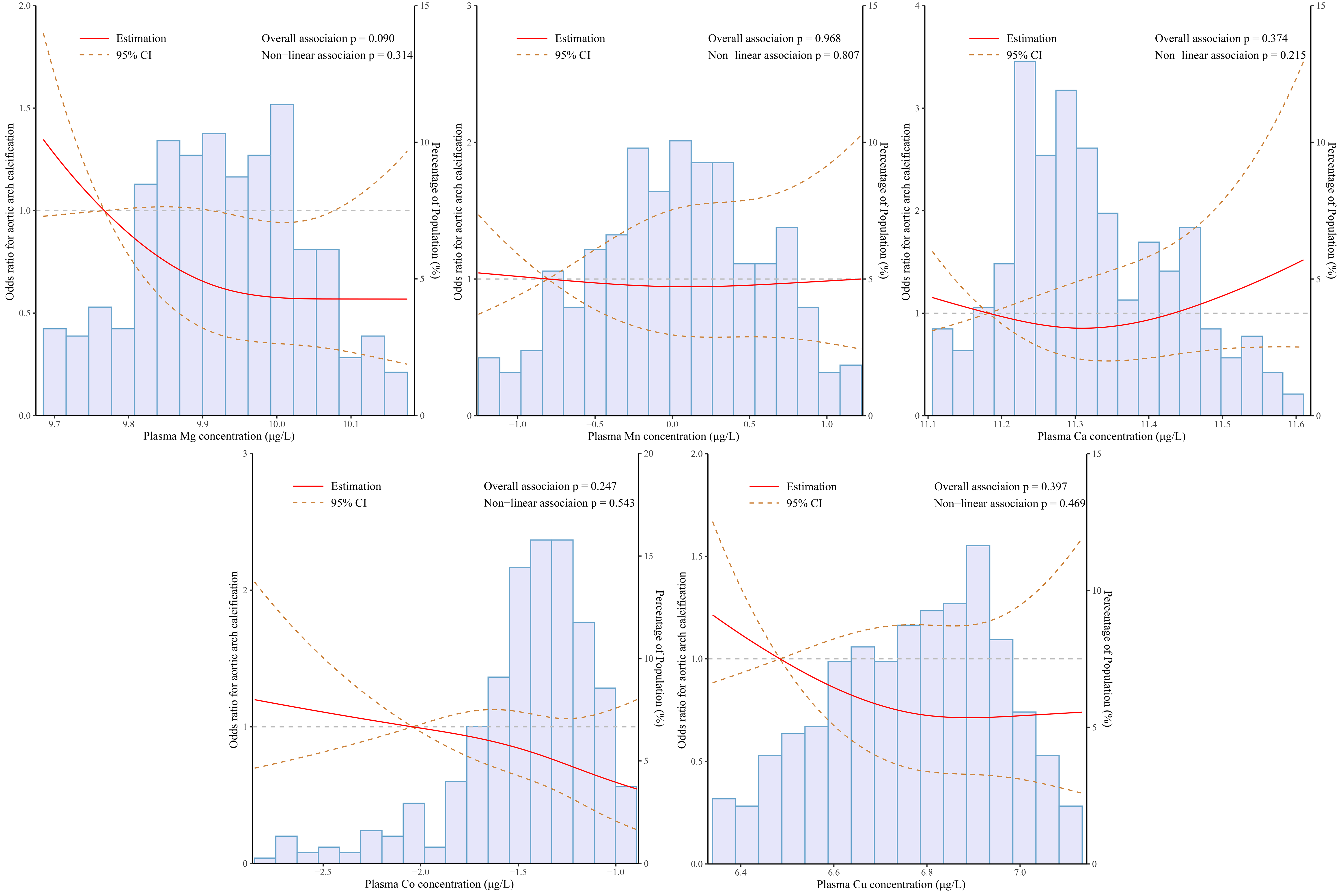


**Figure S6. The restricted cubic spline for the associations between plasma metal concentrations and the risk of AoAC in females.**

Note: The lines represent adjusted odds ratios (solid red lines) and 95% confidence intervals (long dashed lines) based on the restricted cubic spline models for the ln-transformed concentrations of plasma Mg, Mn, Ca, Co and Cu. The reference values were set at the 10th percentiles, and the knots were set at the 10th, 50th and 90th percentiles of the ln-transformed concentrations, respectively. Adjusted factors were consistent with the single-metal model 3. AoAC, aortic arch calcification; Mg, magnesium; Mn, Manganese; Ca, calcium; Co, cobalt; Cu, copper.


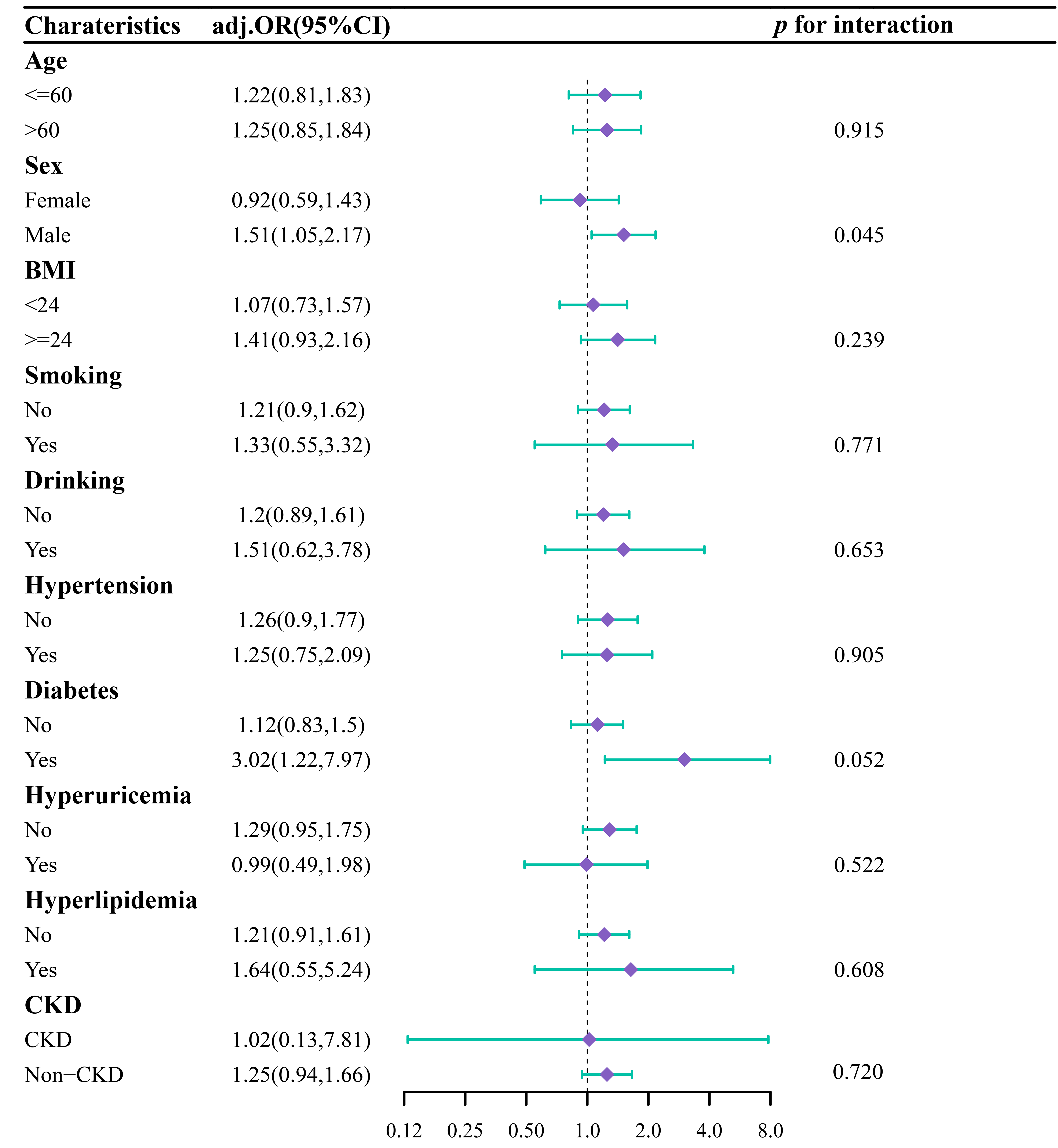


**Figure S7. Subgroup analysis of the association between Mn and AoAC.**

Note: *p* for interaction, *p*-values for the interaction terms. The interaction was examined by adding an interaction term between a specific metal and the stratification variables. AoAC, aortic arch calcification; BMI, body mass index; CKD, chronic kidney disease; Mn, Manganese.


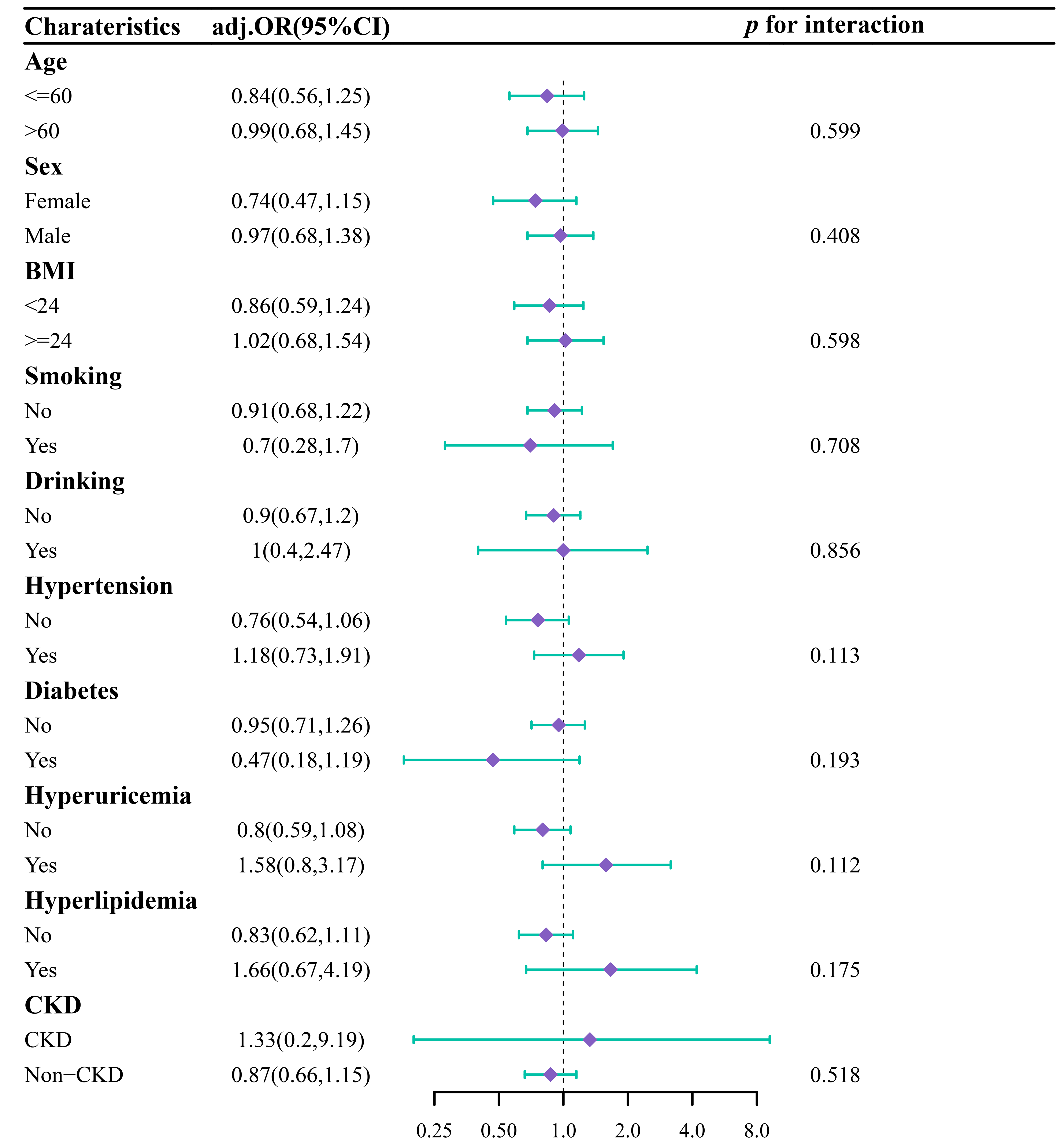


**Figure S8. Subgroup analysis of the association between Mg and AoAC.**

Note: *p* for interaction, *p*-values for the interaction terms. The interaction was examined by adding an interaction term between a specific metal and the stratification variables. AoAC, aortic arch calcification; BMI, body mass index; CKD, chronic kidney disease; Mg, magnesium.


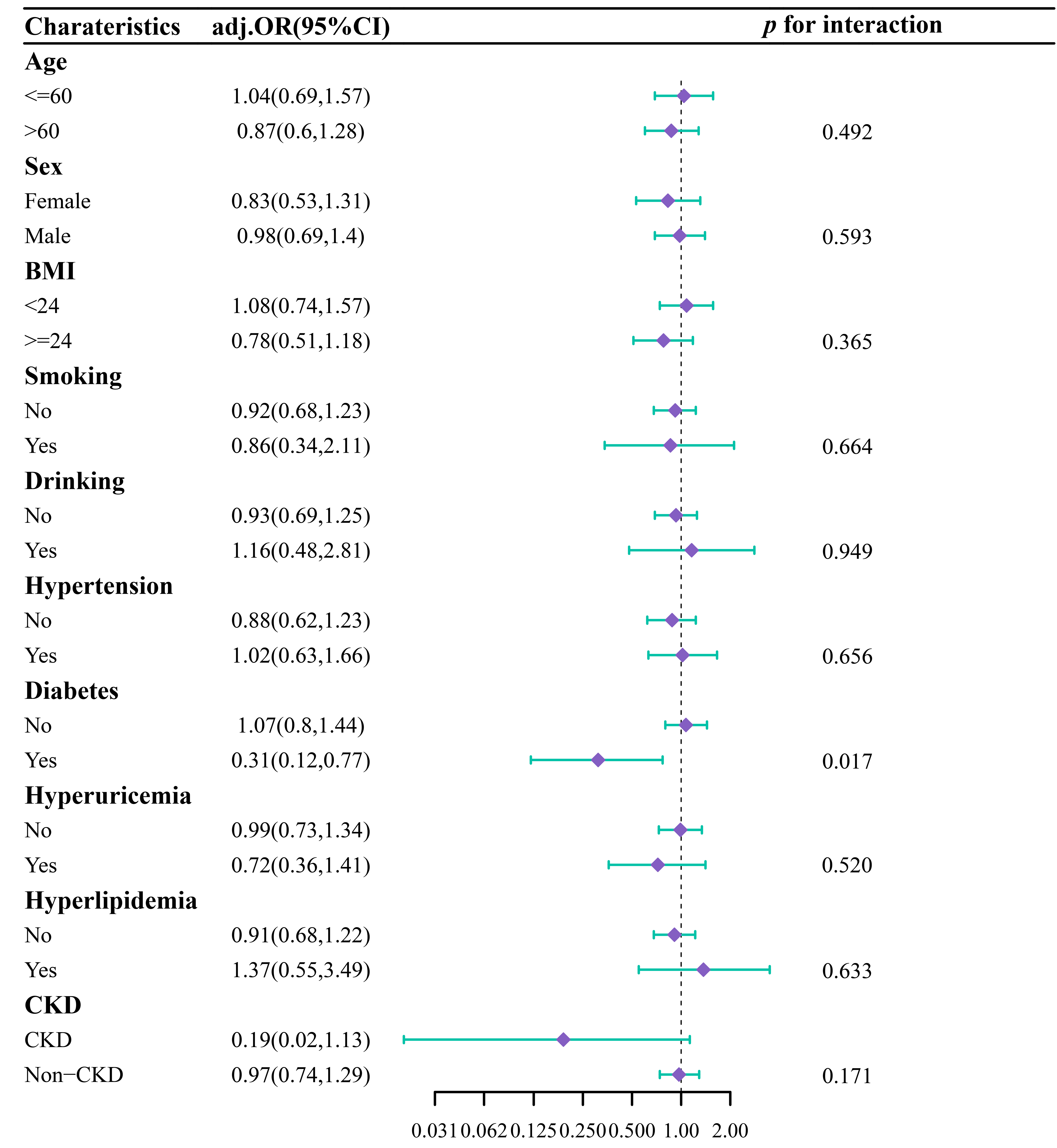


**Figure S9. Subgroup analysis of the association between Cu and AoAC.**

Note: *p* for interaction, *p*-values for the interaction terms. The interaction was examined by adding an interaction term between a specific metal and the stratification variables. AoAC, aortic arch calcification; BMI, body mass index; CKD, chronic kidney disease; Cu, copper.


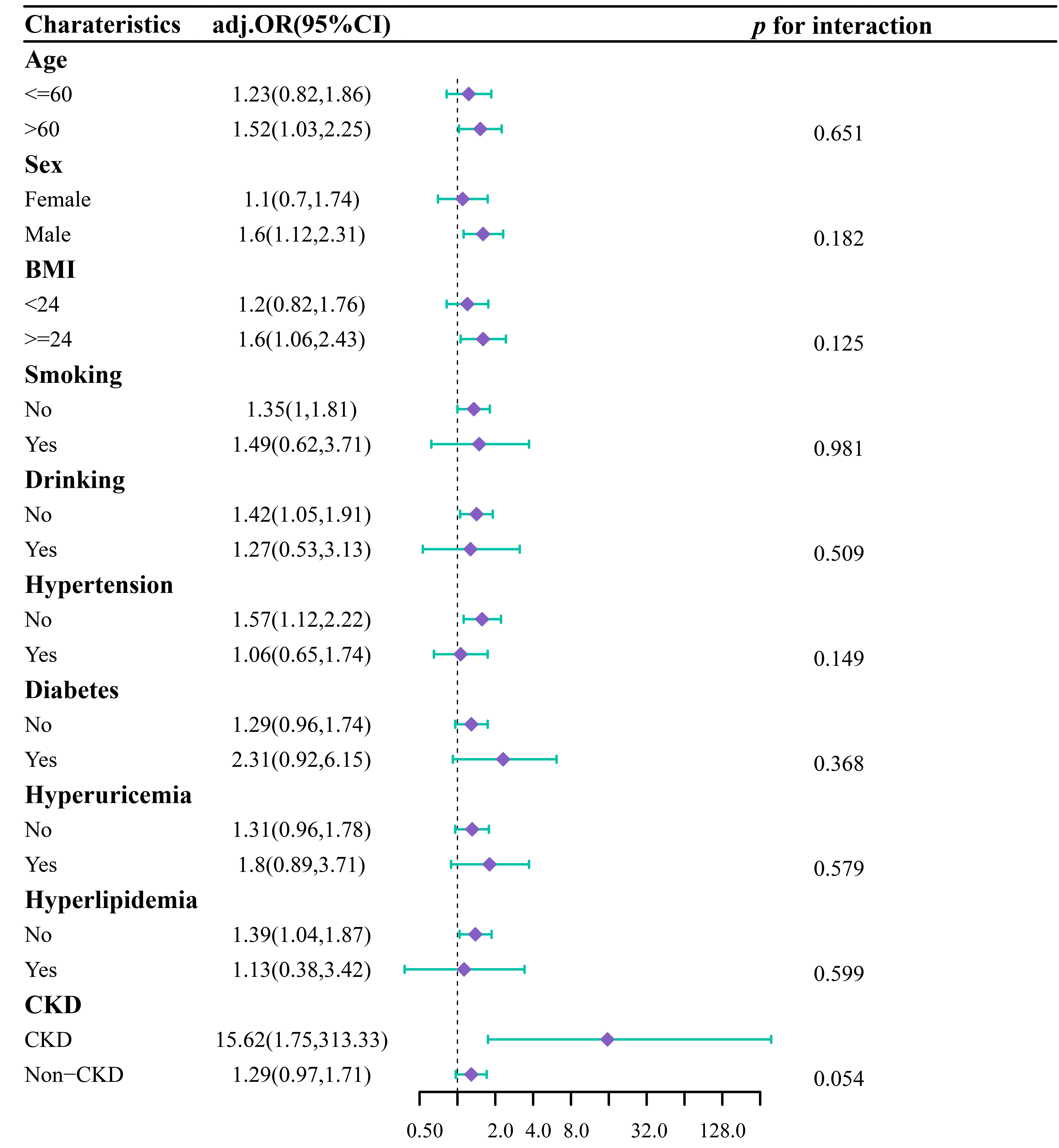


**Figure S10. Subgroup analysis of the association between Ca and AoAC.**

Note: *p* for interaction, *p*-values for the interaction terms. The interaction was examined by adding an interaction term between a specific metal and the stratification variables. AoAC, aortic arch calcification; BMI, body mass index; CKD, chronic kidney disease; Ca, calcium.


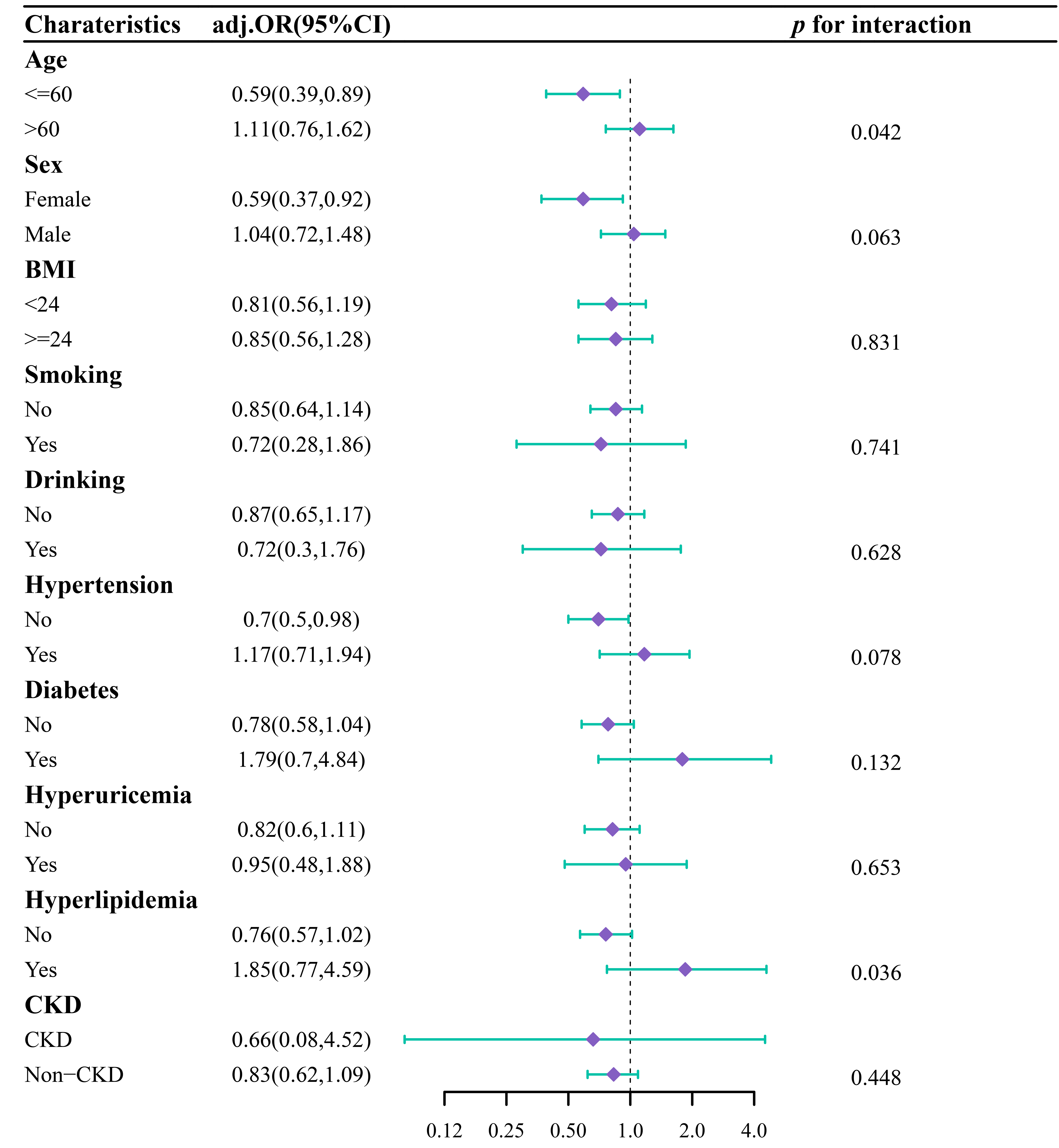


**Figure S11. Subgroup analysis of the association between Co and AoAC.**

Note: *p* for interaction, *p*-values for the interaction terms. The interaction was examined by adding an interaction term between a specific metal and the stratification variables. AoAC, aortic arch calcification; BMI, body mass index; CKD, chronic kidney disease; Co, cobalt.

**
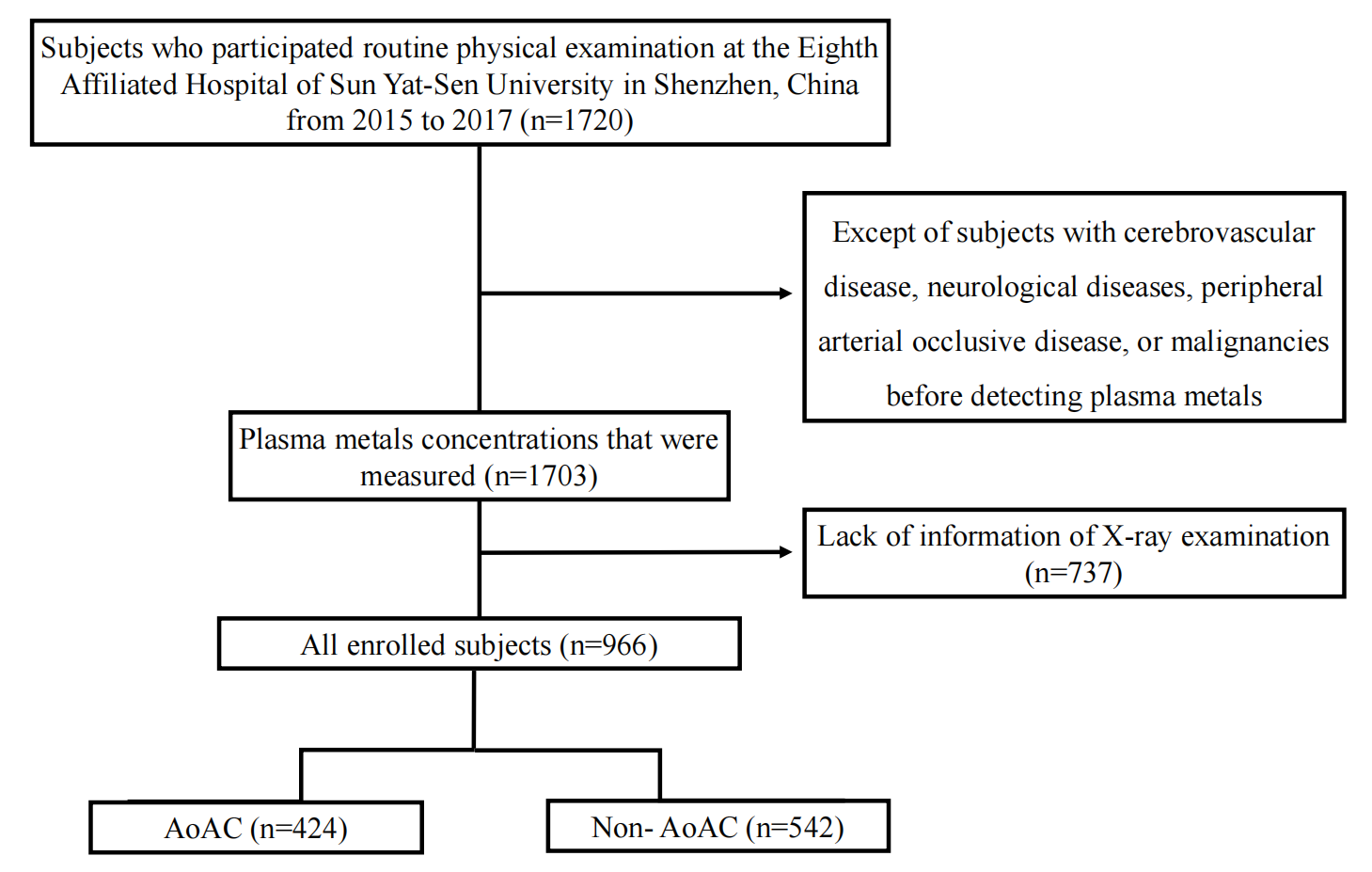
**

**Figure S12. Flowchart of the population enrollment in this study.**

Note: AoAC, aortic arch calcification.
